# Efficient differentiation and purification of human induced pluripotent stem cell-derived endothelial progenitor cells and expansion with the use of inhibitors of ROCK, TGF-β, and GSK3β

**DOI:** 10.1016/j.heliyon.2020.e03493

**Published:** 2020-03-03

**Authors:** Hiromasa Aoki, Misaki Yamashita, Tadahiro Hashita, Koichi Ogami, Shinichi Hoshino, Takahiro Iwao, Tamihide Matsunaga

**Affiliations:** a Department of Clinical Pharmacy, Graduate School of Pharmaceutical Sciences, Nagoya City University, Nagoya, 467-8603, Japan; b Department of Biological Chemistry, Graduate School of Pharmaceutical Sciences, Nagoya City University, Nagoya, 467-8603, Japan

**Keywords:** Human induced pluripotent stem cell, Endothelial progenitor cell, Differentiation, Purification, Expansion, Cell biology, Cell culture, Cell differentiation, Stem cell research, Pharmaceutical science

## Abstract

Endothelial cells (ECs) and endothelial progenitor cells (EPCs) play crucial roles in maintaining vascular health and homeostasis. Both cell types have been used in regenerative therapy as well as in various *in vitro* models; however, the properties of primary human ECs and EPCs are dissimilar owing to differences in genetic backgrounds and sampling techniques. Human induced pluripotent stem cells (hiPSCs) are an alternative cell source of ECs and EPCs. However, owing to the low purity of differentiated cells from hiPSCs, purification via an antigen–antibody reaction, which damages the cells, is indispensable. Besides, owing to limited expandability, it is difficult to produce these cells in large numbers. Here we report the development of relatively simple differentiation and purification methods for hiPSC-derived EPCs (iEPCs). Furthermore, we discovered that a combination of three small molecules, that is, Y-27632 (a selective inhibitor of Rho-associated, coiled-coil containing protein kinase [ROCK]), A 83–01 (a receptor-like kinase inhibitor of transforming growth factor beta [TGF-β]), and CHIR-99021 (a selective inhibitor of glycogen synthase kinase-3β [GSK3β] that also activates Wnt), dramatically stimulated protein synthesis-related pathways and enhanced the proliferative capacity of iEPCs. These findings will help to establish a supply system of EPCs at an industrial scale.

## 1 Introduction

Endothelial cells (ECs) make up the innermost layer of blood vessels and play crucial roles in maintaining vascular health (Esper et al., 2006). Disruption to the vessel endothelium has been associated with various pathological conditions, including arteriosclerosis, heart failure, hypertension, and ischemia (Esper et al., 2006).

Endothelial progenitor cells (EPCs), which are immature ECs, differentiate into mature ECs or release angiogenic factors to form new blood vessels (Goligorsky and Salven, 2013). The transplantation of EPCs was shown to promote regenerative effects in animal models of ischemic diseases and irreversible fibrosis (Kalka et al., 2000; Nakamura et al., 2007). Moreover, a recent clinical study reported that EPC transplantation had therapeutic effects in patients with hypertension and ischemic diseases (Chong et al., 2016). In addition, EPCs and ECs have been applied in various *in vitro* models of pathological diseases (Farcas et al., 2009; Goya et al., 2003), organs-on-chips (Huh et al., 2010) for *in vitro* experiments as an alternative to animal models, and pharmacokinetic models of the blood–brain barrier (Malinovskaya et al., 2016).

However, there are two main problems with the use of ECs and EPCs: (1) human primary EPCs have limited expandability (Igreja et al., 2008) and (2) the properties and characteristics of EPCs are heterogeneous owing to differences in genetic backgrounds and sampling techniques. Especially, the low number and weakened function of EPCs are serious problems for autologous transplantation for patients with lifestyle-related diseases (Esposito et al., 2009; Tepper, 2002).

Human pluripotent stem cells (hPSCs), including human induced pluripotent stem cells (hiPSCs) and human embryonic stem cells, proliferate infinitely and have the ability to differentiate into various cell types (Thomson et al., 1998; Takahashi et al., 2007). Therefore, hPSCs could differentiate into homogeneous cells. To address the problems related to a stable supply and consistent quality, hPSC-derived EPCs are considered as a viable alternative to human primary EPCs. Actually, hPSC-derived ECs and EPCs have been applied in various studies (Jang et al., 2019; Shen et al., 2018).

Protocols for the efficient generation and differentiation of hPSC-derived ECs and EPCs have been recently reported (Aoki et al., 2019; Lian et al., 2014; Nguyen et al., 2016; Zhang et al., 2017a; Sriram et al., 2015). However, several problems exist with the use of hiPSC-derived ECs (iECs) and EPCs (iEPCs) in regenerative medicine and pharmacokinetic evaluation on an industrial scale. Because of the low purity of differentiated iEPCs, purification using cell sorters or magnetic beads is indispensable; however, this process is complicated and damages the cells. Besides, methods for the generation of large numbers of iEPCs from hiPSCs have not been optimized.

We therefore devised a method to easily produce functional high-purity iEPCs on a large scale without requiring cumbersome purification methods. Here, we demonstrate that high-purity iEPCs can be obtained easily and quickly by arranging the treatment time of the dissociation solution at the final stage of differentiation. In contrast to other methods that use cell sorters or magnetic beads, the method described in this study does not require complex manipulations or long incubation times for the antigen–antibody reaction. The obtained iEPCs maintained basic endothelial functions, including tube formation and uptake of acetylated low-density lipoprotein. Furthermore, iEPCs were successfully expanded using a combination of three small molecules, which stimulated cell proliferation of rat hepatocytes (Katsuda et al., 2017), collectively termed YAC: Y-27632 (a selective inhibitor of Rho-associated, coiled-coil containing protein kinase [ROCK]), A 83–01 (a receptor-like kinase inhibitor of transforming growth factor beta [TGF-β]), and CHIR-99021 (a selective inhibitor of glycogen synthase kinase-3β [GSK3β] that also activates Wnt). YAC supplementation dramatically enhanced the proliferative capacity of iEPCs and retained the EPC phenotype during expansion culture. Moreover, RNA-sequencing (RNA-seq) analysis indicated that YAC stimulated mRNA translation and protein synthesis by iEPCs. These results will contribute to the establishment of stable supply systems of functional high-purity and high-quality iEPCs on an industrial scale.

## 2 Materials and methods

### 2.1 Materials

Human iPS cell lines 610B1, 606A1, and 648A1 were purchased from Riken BioResource Center (Tsukuba, Japan). Human umbilical vein endothelial cells (HUVECs) and Endothelial Cell Medium were purchased from ScienCell Research Laboratories, Inc. (Carlsbad, CA, USA). Fibronectin, l-glutamine, a 1:1 mixture of Dulbecco's modified Eagle's medium and Ham's nutrient mixture F-12 (DMEM/F12), MEM non-essential amino acids, l-ascorbic acid phosphate magnesium salt n-hydrate, and hydrocortisone were purchased from Wako Pure Chemical Industries, Ltd. (Osaka, Japan). Porcine skin gelatin, 2-mercaptoethanol, 1-thioglycerol, and GlutaMAX supplement were purchased from Sigma-Aldrich Corporation (St. Louis, MO, USA). Gibco™ KnockOut™ Serum Replacement (KSR), insulin–transferrin–selenium (ITS), TrypLE™ Select cell dissociation reagent, Gibco™ Cell Therapy Systems™ KnockOut™ SR XenoFree medium, Vitronectin-N (VTN-N), chemically defined lipid concentrate, Essential 8™ Flex medium, Gibco™ Human Endothelial Serum-free medium (HE-SFM), Gibco™ Medium 200, and eBioscience™ Flow Cytometry Staining Buffer were purchased from Thermo Fisher Scientific (Waltham, MA, USA). Fibroblast growth factor -2 (FGF2) was purchased from PeproTech, Inc. (Cromwell, CT, USA). Born morphogenetic protein-4 (BMP4) was purchased from ProSpec-Tany TechnoGene Ltd. (Rehovot, Israel). Penicillin–streptomycin solution was purchased from Biological Industries USA, Inc. (Cromwell, CT, USA). Vascular endothelial growth factor (VEGF) was purchased from BioLegend (San Diego, CA, USA). A 83–01 was purchased from Cayman Chemical (Ann Arbor, MI, USA). CHIR-99021 and Y-27632 were purchased from Focus Biomolecules, LLC (Plymouth Meeting, PA, USA). Epidermal growth factor (EGF) was purchased from GenScript Biotech Corporation (Piscataway, NJ, USA). TC Protector was purchased from DS Pharma Biomedical (Osaka, Japan). CELLBANKER® and STEM-CELLBANKER® containing dimethyl sulfoxide (DMSO) were purchased from Nippon Zenyaku Kogyo Co., Ltd. (Fukushima, Japan). Cell Reservoir One containing DMSO was purchased from Nacalai Tesque, Inc. (Kyoto, Japan). An Agencourt RNAdvance Tissue Total RNA Purification Kit was purchased from Beckman Coulter, Inc. (Brea, CA, USA). ReverTra Ace® qPCR RT Master Mix was purchased from Toyobo Co., Ltd. (Osaka, Japan). KAPA SYBR® FAST qPCR Master Mix (2×) was purchased from Nippon Genetics Co., Ltd. (Tokyo, Japan). Corning® Matrigel® Growth Factor Reduced (GFR) Basement Membrane Matrix (Matrigel GFR) was purchased from Corning Incorporated (Corning, NY, USA). 1,1′-Dioctadecyl-3,3,3′,3′-tetramethyl-indocarbocyanine perchlorate acetylated low-density lipoprotein (Dil-Ac-LDL) was purchased from Alfa Aesar (Ward Hill, MA, USA). A CellTiter-Glo® 2.0 Cell Viability Assay kit was purchased from Promega Corporation (Madison, WI, USA). A Cellular Senescence Detection/Quantification Kit–SPiDER-β-Gal was purchased from Dojindo Laboratories (Kumamoto, Japan). Block-Ace solution was purchased from KAC Co., Ltd. (Kyoto, Japan).

### 2.2 Cell culture

The hiPS cell lines 610B1, 606A1, and 648A1 were maintained in Essential 8 Flex medium on VTN-N (1 μg/cm^2^)-coated dishes. iPSCs were passaged using 0.5 mM ethylenediaminetetraacetic acid. HUVECs were cultured in Endothelial Cell Medium in accordance with the manufacturer's instructions.

### 2.3 Differentiation of iPSCs into iEPCs

hiPSCs were dissociated with the use of TrypLE Select cell dissociation reagent, seeded on VTN-N (1 μg/cm^2^)-coated dishes (2 × 10^4^ cells/cm^2^), and cultured in Essential 8 Flex medium supplemented with 10 μM Y-27632 for 24 h. On day 0, the Essential 8 Flex medium was replaced with modified DMEM/F12 (DMEM/F12 containing 0.1% chemically defined lipid concentrate, 0.1 × ITS, 2 mM GlutaMAX, 450 mM 1-thioglycerol, 50 μg/mL l-ascorbic acid phosphate magnesium salt n-hydrate, and 1 × penicillin–streptomycin solution) supplemented with 5 μM CHIR-99021. On day 1, the medium was replaced with modified DMEM/F12 supplemented with 50 ng/mL FGF2. On day 2, the medium was replaced with modified DMEM/F12 supplemented with 50 ng/mL VEGF and 25 ng/mL BMP4, and then replaced daily. On day 5, differentiated cells treated with 10 μM Y-27632 for 1 h were dissociated with TrypLE Select and replated onto porcine skin gelatin (0.1%) or VTN-N (0.25 μg/cm^2^)-coated (for xeno-free conditions) dishes (3.5 × 10^4^ cells/cm^2^) and cultured in EPC medium (HE-SFM containing 5% KSR and 1 × penicillin–streptomycin solution) supplemented with 50 ng/mL VEGF and 10 ng/mL FGF2. For the xeno-free condition, xeno-free EPC medium (Medium 200 containing 5% CTS KnockOut SR XenoFree Medium and 0.1 × ITS) supplemented with 2.5 μM hydrocortisone, 50 ng/mL VEGF, and 10 ng/mL FGF2 was used from day 5 to day 8. The medium was changed daily.

### 2.4 Purification of iEPCs

On day 8, differentiated cells (under both normal and xeno-free conditions) treated with 10 μM Y-27632 for 1 h were washed once with D-phosphate-buffered saline (PBS) (−) and then treated with TrypLE Select for 45–60 s at 37 °C until some of the cells were floating. Then, extra cells were stripped completely by tapping the dish several times. After washing with D-PBS (−) three times (and aspirating the edge of the dish), purified iEPCs were treated with TrypLE Select for 6–12 min at 37 °C, collected in a 15- or 50-mL tube, and centrifuged at 100 × *g* for 5 min. Then, purified iEPCs were resuspended in fresh medium and seeded onto VTN-N (1 μg/cm^2^)-coated dishes (1.5 × 10^4^ cells/cm^2^). In the case of replating of non-purified cells on day 8, differentiated cells treated with 10 μM Y-27632 for 1 h were washed with D-PBS (−) once, treated with TrypLE Select for 7–13 min at 37 °C, collected in a 15- or 50-mL tube, and centrifuged at 100 × *g* for 5 min. Then, the cells were resuspended in fresh medium and seeded onto VTN-N (1 μg/cm^2^)-coated dishes (1.5 × 10^4^ cells/cm^2^). After purification, iEPCs differentiated under normal conditions were cultured in EPC medium supplemented with 10 ng/mL EGF and 20 ng/mL FGF2. For the xeno-free condition, iEPCs were cultured in xeno-free EPC medium supplemented with 2.5 μM hydrocortisone, 50 ng/mL VEGF, and 10 ng/mL FGF2.

### 2.5 Expansion culture of iEPCs

After purification, iEPCs were cultured in EPC medium supplemented with 10 ng/mL EGF, 20 ng/mL FGF2, and DMSO (control group) or 10 μM Y-27632, 0.5 μM A 83–01, and 3 μM CHIR-99021. The medium was changed on the first and third days after seeding. At the time of passage, after washing with D-PBS (−) once, iEPCs were dissociated with the use of TrypLE Select, collected in a 15- or 50-mL tube containing medium, centrifuged at 100 × *g* for 5 min, resuspended in fresh medium, and seeded onto VTN-N (1 μg/cm^2^)-coated dishes at a density of 1.5 × 10^4^ cells/cm^2^. If the number of acquired cells was less than 1.5 times the seeding cell number (610B1-derived iEPCs on day 34 and 606A1-derived iEPCs on day 30), the cells were seeded at a density of 3 × 10^4^ cells/cm^2^. Acquired iEPCs were counted according to the trypan blue method using a Countess™ II Automated Cell Counter (Thermo Fisher Scientific). During the expansion culture, the 610B1-derived cells were passaged on days 11, 14, 17, 20, 24, 29, and 34, and the 606A1-derived cells were passaged on days 11, 14, 17, 20, 25, and 30.

### 2.6 RNA extraction, reverse transcription, and RT-qPCR

Total RNA from HUVECs (passage 3), hiPSCs, and hiPSC-derived cells was purified using an Agencourt RNAdvance Tissue Total RNA Purification Kit. Reverse transcription was performed using ReverTra Ace qPCR RT Master Mix. Relative mRNA expression levels were measured using the KAPA SYBR Fast qPCR Kit with a LightCycler® 96 System (Roche, Basel, Switzerland). mRNA expression levels were normalized to those of hypoxanthine guanine phosphoribosyltransferase 1 (HPRT1). The RT-qPCR primers are listed in Table 1.

**Table 1:** RT-qPCR primer sequences and antibodies for immunofluorescence analysis.

Gene	Forward primer sequence (5′ → 3′)	Reverse primer sequence (5′ → 3′)
*CDH5*	GATTTGGAACCAGATGCACA	ACTTGGCATTCTTGCGACTC
*KDR*	CTGCAAATTTGGAAACCTGTC	GAGCTCTGGCTACTGGTGATG
*vWF*	CGGCTTGCACCATTCAGCTA	TGCAGAAGTGAGTATCACAGCCATC
*PECAM1*	AGTCGGACAGTGGGACGTAT	ATGACCTCAAACTGGGCATC
*CD34*	ATCCTAAGTGACATCAAGGCAGA	TCTCCCCTGTCCTTCTTAAACTC
*HPRT1*	CTTTGCTTTCCTTGGTCAGG	TCAAGGGCATATCCTACAACA
*TBXT*	ACCCAGTTCATAGCGGTGAC	CAATTGTCATGGGATTGCAG
*MMP1*	TACGAATTTGCCGACAGAGA	TCCTTGGGGTATCCGTGTAG
*OCT-4*	AGCGAACCAGTATCGAGAAC	TTACAGAACCACACTCGGAC
*COL4A1*	TACAAGGTGTCATTGGGTTTCC	TGGTTCTCCAGTATCACCCTTT
*COL4A6*	AACAAGTTGTGGCTGCTCCT	TCCTCTCGCTCCTTTCTCAG
*CREB5*	CTGATGATTCATAGGCACAAACA	GCAGTTCTTCAGGAATCTCGTT
*FGFR3*	CTCTGTCGAGCCACCAATTT	GGATGCCTGCATACACACTG
*IGF2*	ATCATCGTCCAGGCAGTTTC	TCTTCGGCCCCTGTACTCTA
*ITGA2*	GAACCGAATGGGAGATGTGTAT	AGGCTCATGTTGGTTTTCATCT
*ITGA8*	GCAACAGTGAAAGCTCACAAAG	CCTTTTCTGGTGTCGGTTTAAG
*ITGA11*	ACGACATCAGTGGCAATAAGTG	ACTTGTACACGTCTCCCGTCTT
*ITGAV*	TTTGATGCAACAGGCAATAGAG	ATCCTGTTTCGACCTCACAGAT
*LAMB3*	CATCTACCTGTGGACTGACCAA	AGTCACACTTGCAGCATTTCAT
*LAMC2*	GACTCCAAGTGTGACTGTGACC	ACCTATCACAGCGTTCTCCAGT
*LAMC3*	ACCTCTGAGTTCAGCGACATCT	CCGGTCTAGAGAGATGAGGAGTT
*NOS3*	GACTGAAGGCTGGCATCTG	ATGTTACTGTGCGTCCACTCTG
*PDGFB*	TCCCGAGGAGCTTTATGAGA	TCATGTTCAGGTCCAACTCG
*PGF*	CTCGGGACGTCTGAGAAGAT	GTACCACTTCCACCTCTGACGA
*PTEN*	CAGCCATCATCAAAGAGATCG	ACGCCTTCAAGTCTTTCTGC
*TLR4*	CTTCTCAACCAAGAACCTGGAC	ATATGCCCCATCTTCAATTGTC

### 2.7 Immunofluorescence analysis

Differentiated cells and HUVECs were fixed for 15 min at room temperature in 4% paraformaldehyde, washed three times with D-PBS (−) containing 10 mM glycine, and then permeabilized in D-PBS (−) containing 0.1% Triton X100 for 25 min. Cells were blocked with 5% donkey serum for 20 min, incubated with primary antibodies (Table 2) for 120 min, and then incubated with secondary antibodies (Table 2) and 1 μg/mL of 4′,6-diamidino-2-phenylindole (DAPI) for 1 h at room temperature. The Operetta High-Content Imaging System (PerkinElmer, Inc., Waltham, MA, USA) was used to observe stained samples. Harmony® high-content analysis software (Harmony software) was used to determine the number of positive cells (Table 3).

**Table 2:** Antibodies for immunofluorescence analysis.

Target	Source	Catalog number	Species	Dilution	Application
CDH5	Santa Cruz	sc-9989	Mouse	1:25	IF
PECAM1	Abcam	ab28364	Rabbit	1:25	IF
FITC-PECAM1	Beckman	IM1431U	Mouse	1:10	FCM
CD34	Novus	NBP2-32932	Mouse	8 μg/mL	IF
PE-CD34	Beckman	IM1459U	Mouse	1:10	FCM
Ki-67	Affymetrix	14-5699-82	Mouse	1:100	IF
GAPDH (HRP)	Wako	015–25473	Mouse	1:1000	WB
NOS3	Santa Cruz	sc-376751	Mouse	1:500	WB
Puromycin	Dako	PEN-MA001	Mouse	1:1000	WB
Anti-rabbit (Alexa Fluor 488)	Fisher Scientific	A-21206	Donkey	1:200	IF
Anti-mouse (Alexa Fluor 568)	Fisher Scientific	A-11004	Goat	1:200	IF
Anti-mouse (HRP)	Abcam	ab6789	Goat	1:2000	WB

Abbreviations: HRP, horseradish peroxidase; IF, immunofluorescense analysis; WB, western blotting. FCM, flow cytometry.

**Table 3:** Operetta High-Content Imaging System settings and values of minimum intensity of positive cells.

Setting	Conditions
Plate type	PerkinElmer Cell Carrier 96
Objective	20× long WD
Opt. mode	Non-confocal
Excitation	100%
Calculate program	Intensity cell Alexa 488, 568 mean – inner cells

Target	PECAM1	CDH5	CD34	Ki-67	Ac-LDL	β-Gal
Figure 2C (610B1)	750	350	200			
Figure 2C (608A1)	850	450	160			
Figure 2C (648A1)	720	300	135			
Figure 2D (610B1)					930	
Figure 2D (606A1)					450	
Figure 2D (648A1)					450	
Figure 3C	1270	550	550			
Figure 3D					700	
Figure 8B						3000
Figure 9E				500		
Figure 9F					500	

### 2.8 Matrigel tube formation assay

Differentiated cells were dissociated with the use of TrypLE Select, replated into the wells of a 24-well plate coated with 300 μL of Matrigel GFR, and incubated in the medium used before replating supplemented with 50 ng/mL VEGF for 20–24 h. Later, the cells were stained with calcein AM fluorescent dye for 30 min at room temperature, and tube formation was observed using an ECLIPSE Ni microscope (Nikon Corporation, Tokyo, Japan). We chose representative images with robust tube-like structures, formed by the smallest numbers of seeded cells (except for the DMSO group on day 20) (on day 8: 7.5 × 10^4^ cells/well; day 14 DMSO group: 1 × 10^5^ cells/well; day 14 YAC group: 5 × 10^4^ cells/well; day 20 DMSO group: 1 × 10^5^ cells/well; day 20 YAC group: 5 × 10^4^ cells/well; day 29 YAC group: 7.5 × 10^4^ cells/well; day 39 YAC group: 7.5 × 10^4^ cells/well). On day 20, cells in the DMSO group did not form tube-like structures at seeding cell densities of 1.25 × 10^5^ and 1.5 × 10^5^ cells/well to the same extent as at a density of 1 × 10^5^ cells/well.

### 2.9 Dil-Ac-LDL uptake assay

Differentiated cells were incubated with 10 μg/mL Dil-Ac-LDL for 5 h, incubated with 10 μg/mL Hoechst 33342 at room temperature for 30 min, and washed four times with medium. The Operetta High-Content Imaging System was used to observe samples, and Harmony software was used to determine the number of Dil-Ac-LDL-positive cells (Table 3).

### 2.10 Freezing–thawing of iEPCs

Differentiated cells on day 11 were resuspended in TC Protector, CELLBANKER®, STEM-CELLBANKER® containing DMSO, or Cell Reservoir One containing DMSO, in accordance with the manufacturer's instructions, and then frozen at −80 °C. After 1 h, the frozen cells were semi-thawed in a 37 °C water bath and completely dissolved in pre-warmed medium. Subsequently, the cells were transferred to a 15-mL tube containing 10 mL of pre-warmed medium and centrifuged at 100 × *g* for 5 min. Later, the cells (2 × 10^4^ cells/cm^2^) were replated onto VTN-N (1 μg/cm^2^)-coated dishes. Other than the cell viability assay, all analyses were conducted on day 14.

### 2.11 Luminescent cell viability assay

The CellTiter-Glo 2.0 assay was conducted in accordance with the manufacturer's instructions. Briefly, iEPCs on day 11 (5 × 10^3^ cells/cm^2^) were seeded into the wells of a 48-well plate and incubated for 72 h with 10 μM Y-27632, 0.5 μM A83-01, 3 μM CHIR-99021, or a combination of these inhibitors. The plated cells were equilibrated to room temperature for 30 min. Next, 300 μL of CellTiter-Glo 2.0 reagent was added to each well, and the plate was shaken for 2 min on an orbital shaker to induce cell lysis. Then, the contents were incubated at room temperature for 10 min. Later, 100-μL aliquots of the cell lysate were transferred to the wells of a white 96-well plate, and luminescent intensity was recorded in auto gain mode using a Synergy HTX Plate Reader (BioTek Instruments, Inc., Winooski, VT, USA).

### 2.12 Analysis of cellular senescence

β-Galactosidase was detected using the Cellular senescence Detection Kit–SPiDER-β-Gal (Doura et al., 2016) in accordance with the manufacturer's instructions. Briefly, differentiated cells on day 17 were seeded into the wells of a 96-well plate, cultured for 3 days, washed once with medium, and reacted in medium (50 μL) supplemented with Barifomycin A1 working solution (diluted 1/1000) for 1 h. Thereafter, medium (50 μL) supplemented with SPiDER β-Gal working solution (1/1000 dilution) and Hoechst 33342 nucleic acid stain (final concentration, 20 μg/mL) was added to each well, and the plate was incubated at 37 °C for 30 min. After incubation, the cells were washed twice with medium and observed with an Operetta High-Content Imaging System. Harmony software was used to determine the number of SPiDER β-Gal-positive cells (Table 3).

### 2.13 RNA-seq

Total RNA from iEPCs was purified using an Agencourt RNAdvance Tissue Total RNA Purification Kit. Pair-end Illumina sequencing and read count estimation/normalization, and analysis of differentially expressed genes were performed by Novogene Bioinformatics Technology Co., Ltd. (Beijing, China). Briefly, after cleaning, the reads were mapped to the *Homo sapiens* (human) genome assembly GRCh37 (hg19) using the TopHat 2.0.9 spliced read mapper for RNA-seq (https://ccb.jhu.edu/software/tophat/index.shtml). Uniquely mapped paired reads were applied for downstream analysis. The per-gene read counts obtained using the HTSeq 0.6.1 tool were converted to fragments per kilobase per million reads (FPKM). For analysis of differentially expressed genes, the read counts were adjusted with the use of edgeR and then analyzed using the DEGSeq R package (1.12.0). Probability (*p*) values and false discovery rates were adjusted using the Benjamini–Hochberg (BH) method. Genes with an adjusted false discovery rate of <0.05 were extracted as significantly differentially expressed (Tables S1 and S2). Gene Ontology (GO) and Kyoto Encyclopedia of Genes and Genomes (KEGG) enrichment analyses (dotplots and cnetplots) were performed using the clusterProfiler R package (Yu et al., 2012). The following settings were used for GO enrichment analysis: OrgDb = org.Hs.eg.db, keyType = “ENSEMBL.” ont = “ALL,” pAdjustMethod = “BH,” qvalueCutoff = 0.05. The following settings were used for KEGG enrichment analysis: organism = “hsa,” pAdjustMethod = “BH,” qvalueCutoff = 0.05. The RNA-seq data utilized in this study have been deposited in NCBI Gene Expression Omnibus (GEO accession number: GSE138225).

### 2.14 Western blot analysis

Differentiated cells on day 13 were lysed in 1 × sodium dodecyl sulfate–polyacrylamide gel electrophoresis (SDS-PAGE) sample buffer. The protein samples were separated by SDS-PAGE, transferred to polyvinylidene fluoride membranes, which were blocked with 4% Block-Ace solution, washed with tris-buffered saline containing 0.1% Tween 20 (TBS-T), incubated overnight with primary antibodies (Table 2) at 4 °C, and then incubated with secondary antibodies at room temperature for 1 h (Table 2). After washing with TBS-T, Protein bands were detected using an Amersham Imager 600 system (GE Healthcare Life Sciences, Chicago, IL, USA). The protein bands were quantified using Amersham Imager 600 analysis software. Background signals were removed with the Rolling Ball algorithm (Amersham Imager 600 analysis software).

### 2.15 Puromycin incorporation assay

The puromycin incorporation assay was performed as described in a previous study (Ogami et al., 2017). iEPCs were treated with DMSO or YAC from day 11 to day 13. On day 13, iEPCs were treated with 1 μg/mL puromycin for 30 min to label nascent polypeptides. Cycloheximide treatment (10 μg/mL) was performed 10 min before puromycin treatment. Puromycilated proteins were detected by western blot analysis, and the protein bands were quantified using Amersham Imager 600 analysis software. The background signals were subtracted from the band intensities.

### 2.16 Flow cytometric analysis

Differentiated cells were centrifuged at 100 × g for 5 min and fixed with 1 mL of 4% paraformaldehyde for 10 min at room temperature. The cells were then centrifuged at 100 × g for 5 min and permeabilized with 1 mL of cold methanol for 10 min at room temperature. The fixed cells were centrifuged at 100 × g for 5 min, resuspended in 5 mL of Flow Cytometry Staining Buffer, and centrifuged at 100 × g for 5 min. The cells were rotated with antibodies (Table 2) in 50 μL of Flow Cytometry Staining Buffer for 1 h at 4 °C, centrifuged at 100 × g for 5 min at 4 °C, washed, and resuspended in 500 μL of Flow Cytometry Staining Buffer. Finally, the stained cells were analyzed by CytoFLEX (Beckman Coulter, Inc.).

### 2.17 Statistical analyses

For the experiments involving iEPCs, ‘‘n’’ represents the number of iPS cells or differentiated cells on independent dishes or wells, with the exception of partial analyses, such as the luminescent viability assay. Each experiment was repeated at least twice. Data are presented as mean ± standard deviation (SD). The two-tailed Student's *t*-test was used for comparisons between two groups, and one-way analysis of variance followed by Tukey's HSD test or the Games–Howell test was used for comparisons of three or more groups. Levene's test was used to assess the equality of variances. All statistical analyses were performed using SPSS statistics version 25.0 (IBM Japan, Ltd., Tokyo, Japan). A *p* value of <0.05 was considered to be statistically significant.

## 3 Results

### 3.1 Simple differentiation and purification of iEPCs

**Figure 1:**
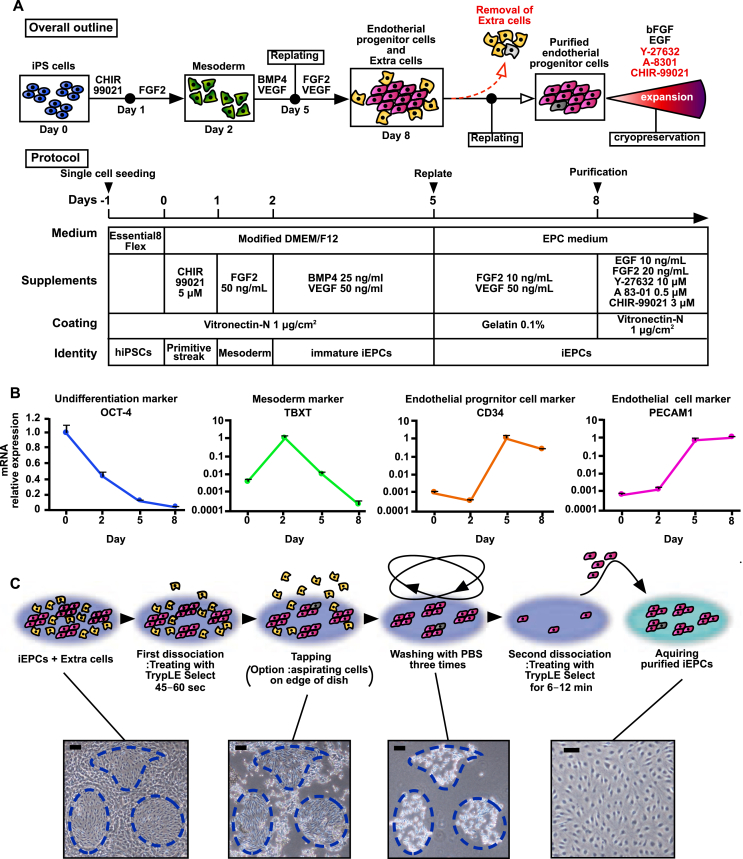
Differentiation and purification scheme of iEPCs. (A) Overall outline and protocols for the differentiation, purification, and expansion of iEPCs. (B) Relative mRNA expression levels of OCT-4, TBXT, CD34, and PECAM1 in 610B1-derived cells were analyzed. The values are normalized to those of HPRT1. The relative mRNA expression levels in hiPS 610B1 cells on day 0 were defined as 1. Data are presented as mean ± SD (*n* = 3). (C) Outline of the quick purification scheme of iEPCs derived from hiPS 610B1 cells on day 8. Scale bars = 100 μm.

In this study, simple and robust methods were used to generate, purify, and expand iEPCs (Figure 1A). The hiPSCs were driven toward differentiation to endothelial lineage cells using a simple protocol improved by combining the methods described in previous reports (Sriram et al., 2015; Nguyen et al., 2016) and those developed in our laboratory. The RT-qPCR results confirmed that the mRNA expression level of the undifferentiation marker octamer-binding transcription factor 4 (OCT-4) had decreased with time, whereas that of the mesodermal marker T-box transcription factor T (TBXT) peaked on day 2 (Figure 1B). Further, the relative expression levels of the EPC marker CD34 and the mature EC marker platelet endothelial cell adhesion molecule (PECAM1) had increased with time (Figure 1B). The results of this simple protocol indicated that hiPSCs were differentiated into endothelial lineage cells.

On day 8, a mixture of more than two cell types (i.e., spindle-shaped cells, iEPCs, and others) with clearly different morphologies from the endothelial lineage cells was observed (Figure 1C). We hypothesized that the adhesion capacity of iEPCs to the culture dish differed from that of extra cells. To acquire only iEPCs, the extra cells were dissociated and removed. Then, the remaining iEPCs were dissociated and replated in new dishes. The iEPCs and extra cells were treated with gentle dissociation solution (TrypLE Select) for 45–60 s at 37 °C, and the dish was tapped several times to completely detach any remaining cells. After washing three times with PBS, the adhered iEPCs were dissociated with TrypLE Select and replated in new dishes (Figure 1C).

### 3.2 Characterization and function of iEPCs

**Figure 2:**
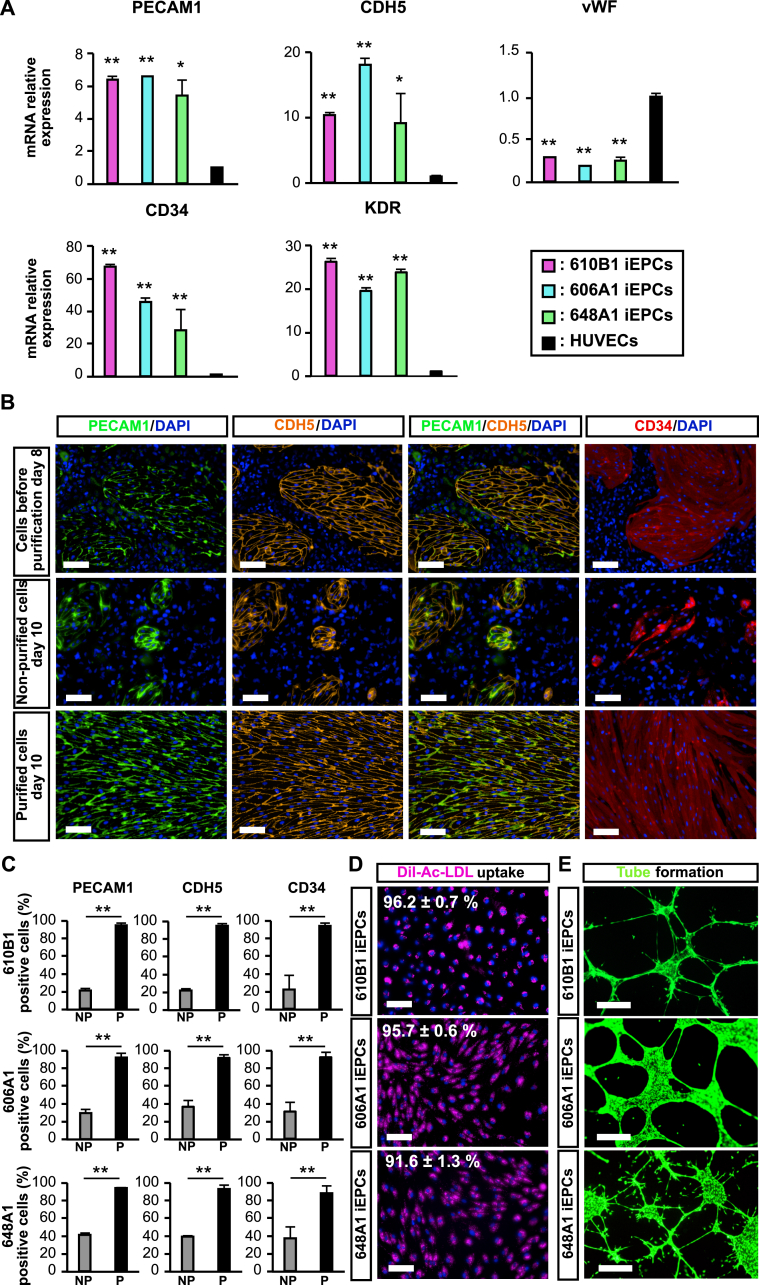
Characterization of purified iEPCs. (A) Relative mRNA expression levels of PECAM1, CDH5, CD34, KDR, and vWF in purified iEPCs on day 8 derived from three hiPS cell lines and HUVECs. The values are normalized to those of HPRT1. The relative mRNA expression levels in HUVECs were defined as 1. Data are presented as mean ± SD (*n* = 3; ∗*p* < 0.05, ∗∗*p* < 0.01; Tukey's HSD test or the Games–Howell test; HUVECs vs. others). (B) Immunofluorescence analyses of PECAM1 (green), CDH5 (orange), and CD34 (red) in differentiated cells just before purification on day 8, non-purified cells on day 10, and purified cells on day 10 derived from hiPS 610B1 cells. DAPI = blue. Scale bars = 100 μm. (C) Positive cell ratio (%) of PECAM1, CDH5, and CD34 in non-purified cells and purified cells (P) on day 10 derived from three hiPS cell lines. Data are presented as mean ± SD (*n* = 3; 6 fields/well; ∗∗*p* < 0.01; Student's *t*-test). (D) Uptake of Dil-Ac-LDL by purified iEPCs on day 10 derived from three hiPS cell lines. Dil-Ac-LDL = pink; Hoechst 33342 = blue. Scale bars = 100 μm. Data are presented as mean ± SD (*n* = 3, 6 fields/well). (E) Matrigel tube formation assay of purified iEPCs on day 8 derived from three hiPS cell lines. Calcein = green. Scale bars = 500 μm.

The gene expression patterns of iEPCs and HUVECs, as determined by RT-qPCR, were compared. The RT-qPCR results confirmed that iEPCs derived from three different iPS cell lines exhibited high expression levels of the typical EC markers PECAM1 and cadherin 5 (CDH5, also known as VE-cadherin), and the EPC markers CD34 and kinase insert domain receptor (KDR, also known as VEGF receptor 2), as compared with HUVECs (Figure 2A). iEPCs had lower expression levels of von Willebrand factor (vWF), a typical EC marker, as compared with HUVECs.

The protein expression levels of the EC markers and the purity of non-purified and purified cells (iEPCs) were examined by immunofluorescence analysis. PECAM1 and CDH5 were localized on the cell membrane, whereas CD34 was in the cytosol of iEPCs (Figure 2B). However, the extra cells did not express such EC markers. Approximately 90% of the purified cells and less than 50% of the non-purified cells derived from the three different iPS cell lines were positive for PECAM1, CDH5, and CD34 (Figure 2C).

The Dil-Ac-LDL uptake assay was conducted to determine whether iEPCs maintained the basic functions of ECs. The results showed that approximately 90% of iEPCs (purified cells) derived from the three iPS cell lines were able to take up Dil-Ac-LDL (Figure 2D). Moreover, the results of the tube formation assay confirmed the presence of tube-like structures (Figure 2E).

**Figure 3:**
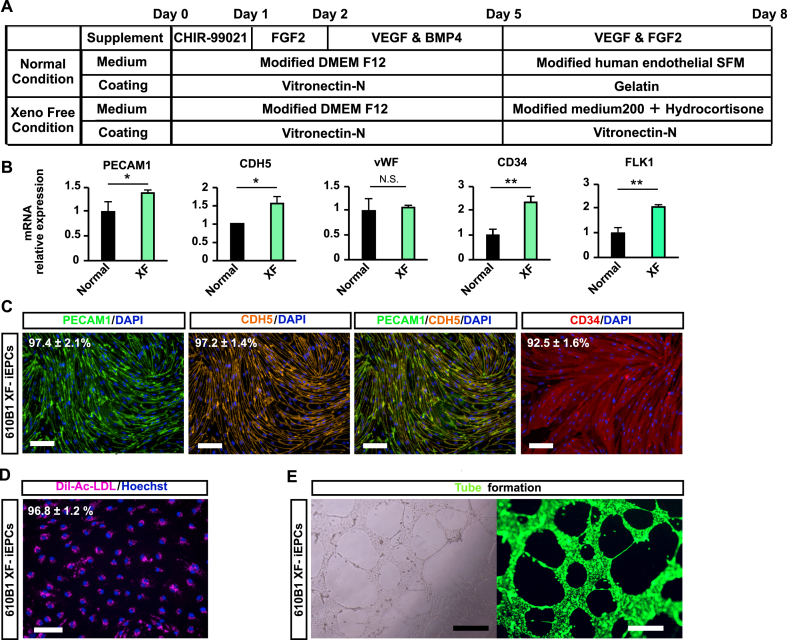
Differentiation and purification of 610B1-derived iEPCs under xeno-free conditions. (A) Differentiation scheme under the xeno-free condition. (B) Comparison of mRNA expression levels between the normal and xeno-free (XF) conditions. Data are presented as mean ± SD (*n* = 3, 6 fields/well; ∗*p* < 0.05; ∗∗*p* < 0.01; N.S. = not significant; Student's *t*-test). (C) Immunofluorescence staining of PECAM1 (green), CDH5 (orange), and CD34 (red) in purified iEPCs differentiated under the XF condition (XF-iEPCs) on day 10. Scale bars = 100 μm. Positive cell ratio (%) of PECAM1, CDH5, and CD34. Data are presented as mean ± SD (*n* = 3). (D) Uptake of Dil-Ac-LDL by XF- iEPCs on day 10. Dil-Ac-LDL = pink; Hoechst 33342 = blue. Scale bars = 100 μm. Data are presented as mean ± SD (*n* = 3, 6 fields/well). (E) Matrigel tube formation assay of XF-iEPCs on day 10. Calcein = green. Scale bars = 500 μm.

iEPCs were successfully differentiated and purified under the xeno-free condition, which is more suitable for regenerative therapy (Figures 3A, 3B, and 3C). Moreover, iEPCs generated under the xeno-free condition maintained EC function (Figures 3D and 3E). These findings confirmed that this method can be adapted for use in the xeno-free condition.

### 3.3 Expansion of iEPCs

**Figure 4:**
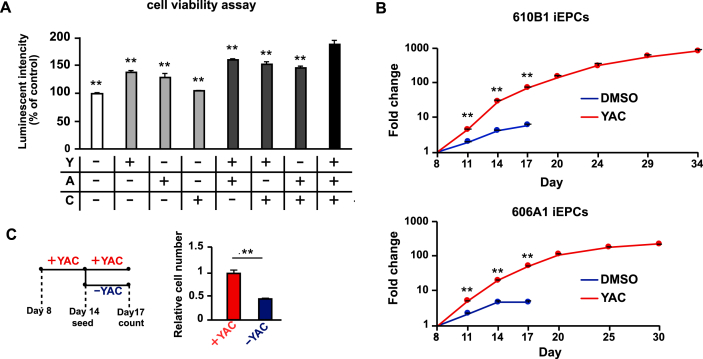
Analyses of the effect of a combination of Y-27632, A 83–01, and CHIR-99021 on iEPCs. (A) The CellTiter-Glo 2.0 assay was performed to analyze the effect of Y-27632 (Y), A 83–01 (A), and CHIR-99021 (C), or their combinations on 610B1-derived iEPCs from day 11 to day 14. Data are presented as mean ± SD (*n* = 6; ∗∗*p* < 0.01; Tukey's HSD test; YAC vs. others). (B) Comparison of the proliferative capacity between DMSO-treated and YAC-treated groups of iEPCs derived from two different iPS cell lines by the trypan blue method. Data are presented as mean ± SD (*n* = 3; ∗∗*p* < 0.01; Tukey's HSD test). (C) Comparison of the cell proliferative capacity of the YAC- (days 8–14) and DMSO-treated (days 14–17) groups and the YAC-treated (days 8–17) group derived from 610B1. The cell numbers of both the groups were confirmed by the trypan blue method. Data are presented as mean ± SD (*n* = 3; ∗∗*p* < 0.01; Student's *t*-test).

After purification, iEPCs proliferated only about twofold in 3 days with supplementation of FGF2 and EGF. Therefore, the iEPCs were expanded by adding small molecule compounds with less lot-to-lot variation as compared with recombinant proteins. Reportedly, Y-27632 enhances the proliferation of human embryonic stem cell-derived ECs (Joo et al., 2012), and SB 43152 (TGF-β inhibitor) enhances the proliferation of hiPSC-derived ECs (James et al., 2010). Furthermore, activation of the Wnt signaling pathway increases the proliferation of human ECs (Masckauchan et al., 2005). Besides, the combination of Y-27632, A 83–01, and CHIR-99021 enhances the proliferation of rat hepatocytes (Katsuda et al., 2017). Hence, combinations of small molecules were tested to determine the best one for proliferation of iEPCs. The results showed that the highest cell viability of iEPCs was achieved with the combination of Y-27632, A 83–01, and CHIR-99021 (YAC) (Figure 4A).

**Figure 5:**
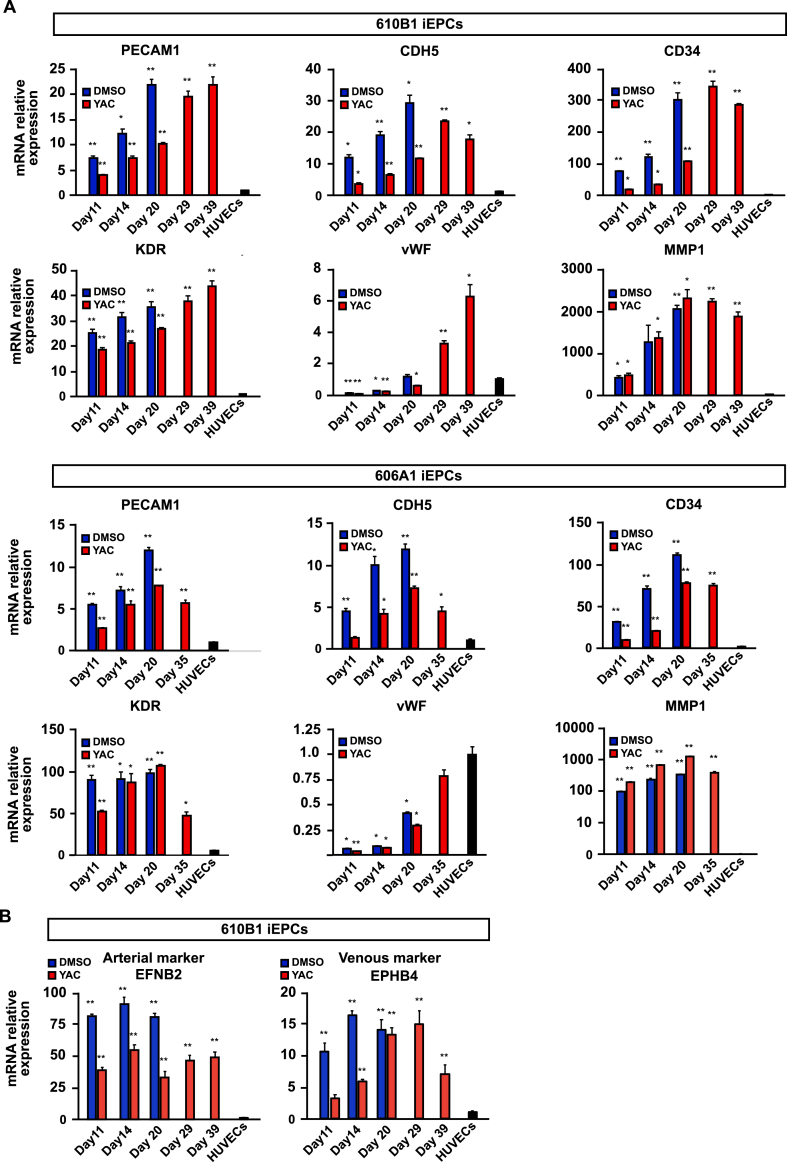
Analyses of gene expression levels of iEPCs during expansion culture. (A) Relative mRNA expression levels of PECAM1, CDH5, CD34, KDR, vWF, and MMP1 of iEPCs derived from two different iPS cell lines and HUVECs. The relative mRNA expression levels in HUVECs were defined as 1. Data are presented as mean ± SD (*n* = 3; ∗*p* < 0.05, ∗∗*p* < 0.01; Tukey's HSD test or the Games–Howell test; HUVECs vs. others). (B) Relative mRNA expression levels of EFNB2 and EPHB4 in 610B1-derived iEPCs in the expansion culture and HUVECs. The relative mRNA expression levels of HUVECs were defined as 1. Data are presented as mean ± SD (*n* = 3; ∗∗*p* < 0.01; Tukey's HSD test or the Games–Howell test; HUVECs vs. others).

**Figure 6:**
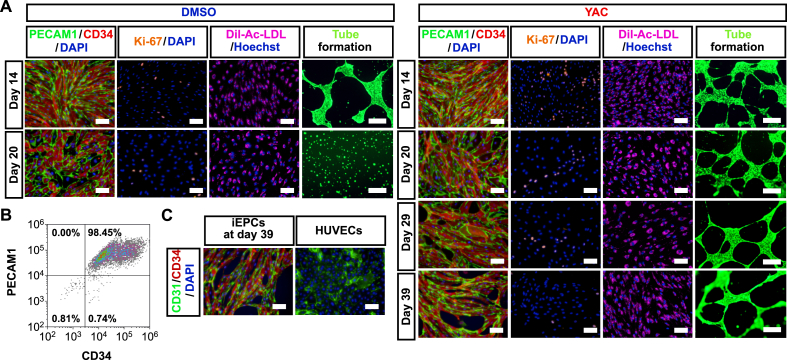
Characterization of iEPCs during expansion culture. (A) Immunofluorescence analyses of PECAM1 (green), CD34 (red), Ki-67 (orange), and Dil-Ac-LDL uptake and tube formation assays of 610B1-derived iEPCs. DAPI = blue (immunofluorescence analyses); Hoechst 33342 (Hoechst) = blue; Dil-Ac-LDL = pink; calcein = green. Scale bars = 100 μm (immunofluorescence analyses and Dil-Ac-LDL uptake assay) and 500 μm (tube formation assay). (B) Flow cytometric analysis of YAC-treated iEPCs at day 14. (C) Immunofluorescence analyses of CD34 (red) in 610B1-derived iEPCs on day 39 and in HUVECs. DAPI = blue. Scale bars = 100 μm.

**Figure 7:**
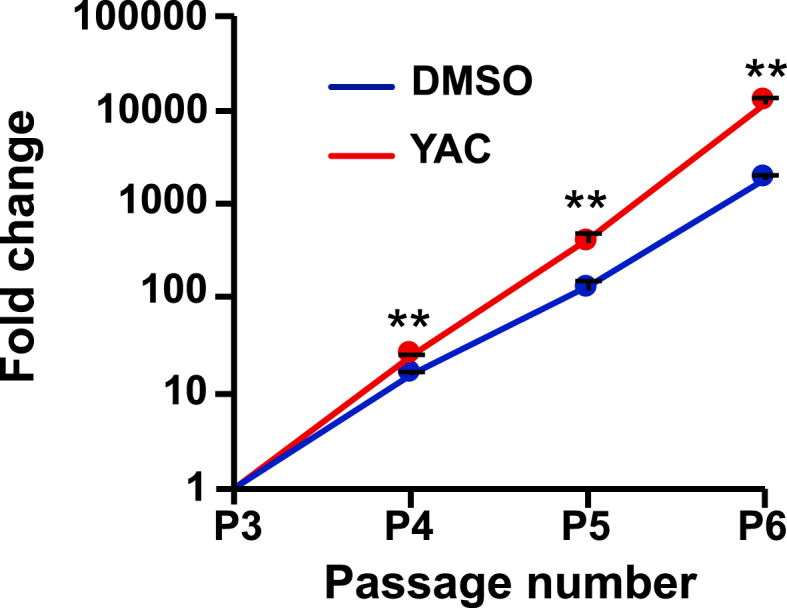
Analyses of the effect of YAC on HUVECs. Comparison of the cell proliferative capacity of DMSO-treated vs. YAC-treated HUVECs in the expansion culture. Data are presented as mean ± SD (*n* = 3; ∗∗*p* < 0.01; Tukey's HSD test).

**Figure 8:**
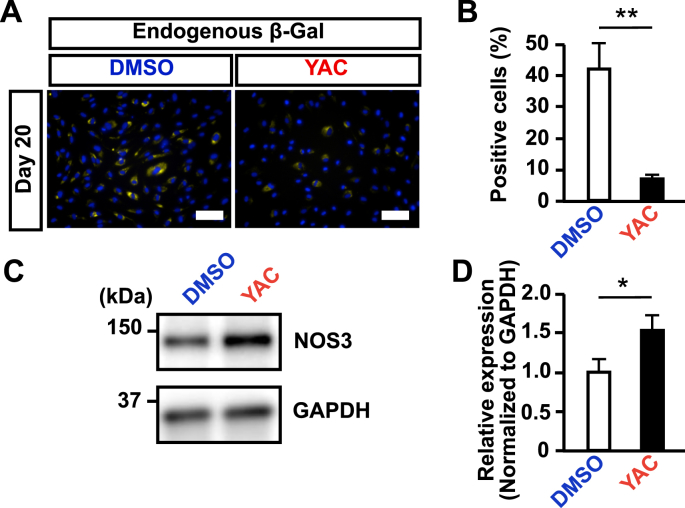
Analyses of cellular senescence. (A and B) β-Galactosidase detection assay of 610B1-derived iEPCs on day 20. Scale bars = 100 μm. Data are presented as mean ± SD (*n* = 3; ∗∗*p* < 0.01; Student's *t*-test). (C and D) NOS3 protein expression levels in 610B1-derived iEPCs treated with DMSO or YAC (treatment from day 11 to day 13) were subjected to western blot analysis. NOS3 levels were normalized to GAPDH levels (*n* = 3; ∗*p* < 0.05; Student's *t*-test). Uncropped and non-adjusted versions of the immunoblotting data are shown in Fig. S2A.

The DMSO-treated (control) group and the YAC-treated group were subcultured until cell proliferation had ceased (acquired cell number <1.5 × seeding cell number), and the cell proliferation rates of the YAC-treated group and the control group were compared. The results showed that the proliferation of the YAC-treated group derived from two different iPS cell lines was significantly increased as compared with the control group (Figure 4B). When YAC was removed, the proliferative capacity was lost instantaneously (Figure 4C). This result indicates that iEPCs stimulated by YAC do not possess autonomous (independent of proliferation stimulus) proliferation, as do cancer cells. To determine whether expanded iEPCs retained the characteristics of ECs, the mRNA expression profiles of both groups of iEPCs and HUVECs were examined. During expansion culture, PECAM1 and CDH5 were strongly expressed by both iEPC groups derived from two different iPS cell lines (Figure 5A). Contrary to our expectations, both groups of iEPCs maintained high expression levels of the markers of immature cells (CD34 and KDR) as compared with HUVECs during expansion culture. The mRNA expression levels of vWF and matrix metalloproteinase 1 (MMP1), a marker of angiogenesis, had gradually increased in both groups of iEPCs during expansion culture. Reportedly, iECs have both arterial and venous phenotypes, and exhibit plasticity characteristics (Olmer et al., 2018). Arterial and venous markers were more highly expressed in iEPCs generated by our protocol than in HUVECs (Figure 5B). To confirm whether iEPCs retain the characteristics of EPCs after expansion culture, the protein expression levels of PECAM1, CD34, and Ki-67 (cell proliferation marker), Dil-Ac-LDL uptake, and tube formation were analyzed. The protein expression profiles of PECAM1, CD34, and Ki-67, and EC functions were retained in YAC-treated iEPCs during expansion culture (Figure 6A), whereas the control group lost the ability to form tube-like structures on day 20. Consistent with these observations, flow cytometric analysis confirmed that ≥95% of the YAC-treated iEPCs were PECAM1/CD34 double positive cells (Figure 6B). Expanded iEPCs also had higher protein expression levels of CD34 than HUVECs (Figure 6C). Besides, YAC enhanced the proliferative capacity of not only iEPCs but also HUVECs (Figure 7). Additionally, analysis of β-galactosidase, an indicator of cellular senescence, in iEPCs indicated that YAC supplementation dramatically suppressed cellular senescence during expansion culture (Figures 8A and 8B). Moreover, the results of western blot analysis confirmed that YAC-treated iEPCs highly expressed nitric oxide synthase 3 (NOS3, also known as endothelial NOS), which prevents EC senescence (Hayashi et al., 2008) (Figures 8C and 8D).

### 3.4 YAC-treated iEPCs were less susceptible to damage induced by freezing–thawing

**Figure 9:**
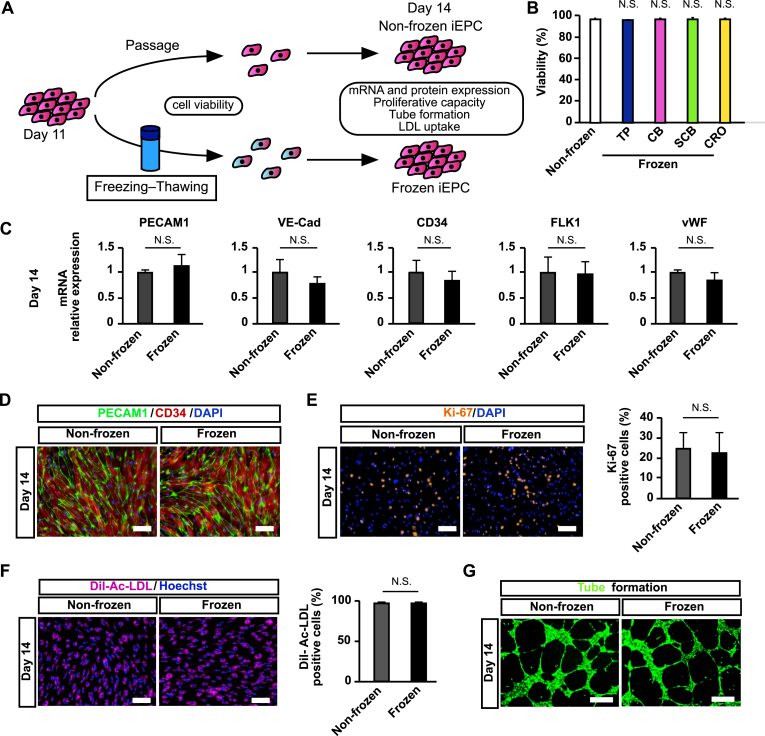
The influence of freezing–thawing on iEPCs. (A) Analyses scheme of the influence of freezing–thawing on iEPCs. (B) Viabilities of thawing of 610B1-derived iEPCs on day 11 frozen by TC Protector (TP), CELLBANKER (CB), STEM-CELLBANKER (SCB), and Cell Reservoir One (CRO). Viabilities were confirmed by the trypan blue method. Data are presented as mean ± SD (*n* = 3; N.S. = not significant; Tukey's HSD test; non-frozen vs. others). (C) Comparison of mRNA expression levels between non-frozen and frozen 610B1-derived iEPCs on day 14 by RT-qPCR. Data are presented as mean ± SD (*n* = 3; N.S. = not significant; Student's *t*-test). (D) Comparison of the protein expression levels of PECAM1 (green) and CD34 (red) between non-frozen and frozen 610B1-derived iEPCs on day 14 by immunofluorescence analysis. DAPI = blue. Scale bars = 100 μm. (E) Immunofluorescence analysis of Ki-67 (orange). Positive cell ratio (%) of Ki-67 in non-frozen and frozen 610B1-derived iEPCs on day 14. DAPI = blue. Scale bars = 100 μm. Data are presented as mean ± SD (*n* = 3, 6 fields/well; N.S. = not significant; Student's *t*-test). (F) Uptake of Dil-Ac-LDL by non-frozen and frozen 610B1-derived iEPCs on day 14. Positive cell ratio (%) of Dil-Ac-LDL in non-frozen and frozen 610B1-derived iEPCs on day 14. Dil-Ac-LDL = pink; Hoechst 33342 = blue. Scale bars = 100 μm. Data are presented as mean ± SD (*n* = 3, 6 fields/well; N.S. = not significant; Student's *t*-test). (G) Matrigel tube formation assay of non-frozen and frozen 610B1-derived iEPCs on day 14. Calcein = green. Scale bars = 500 μm.

Because cellular cryopreservation is indispensable for a stable supply, the influence of freezing–thawing on YAC-treated iEPCs was analyzed (Figure 9A). First, when cell viability was compared between the non-frozen and frozen groups, no significant difference was observed (Figure 9B). Furthermore, there were no significant differences in the gene expression levels of EC and EPC markers between the non-frozen and frozen groups (Figure 9C). In visual observation, the protein expression levels of PECAM1 and CD34 of the frozen group were almost similar to those of the non-frozen group (Figure 9D). There was no significant difference in the number of Ki-67 positive cells (Figure 9E) as well as in the uptake ability of Dil-Ac-LDL between the two groups (Figure 9F). In visual observation, the tube formation ability of the frozen group was almost similar to that of the non-frozen group (Figure 9G).

### 3.5 YAC activates protein synthesis-related pathways in iEPCs

**Figure 10:**
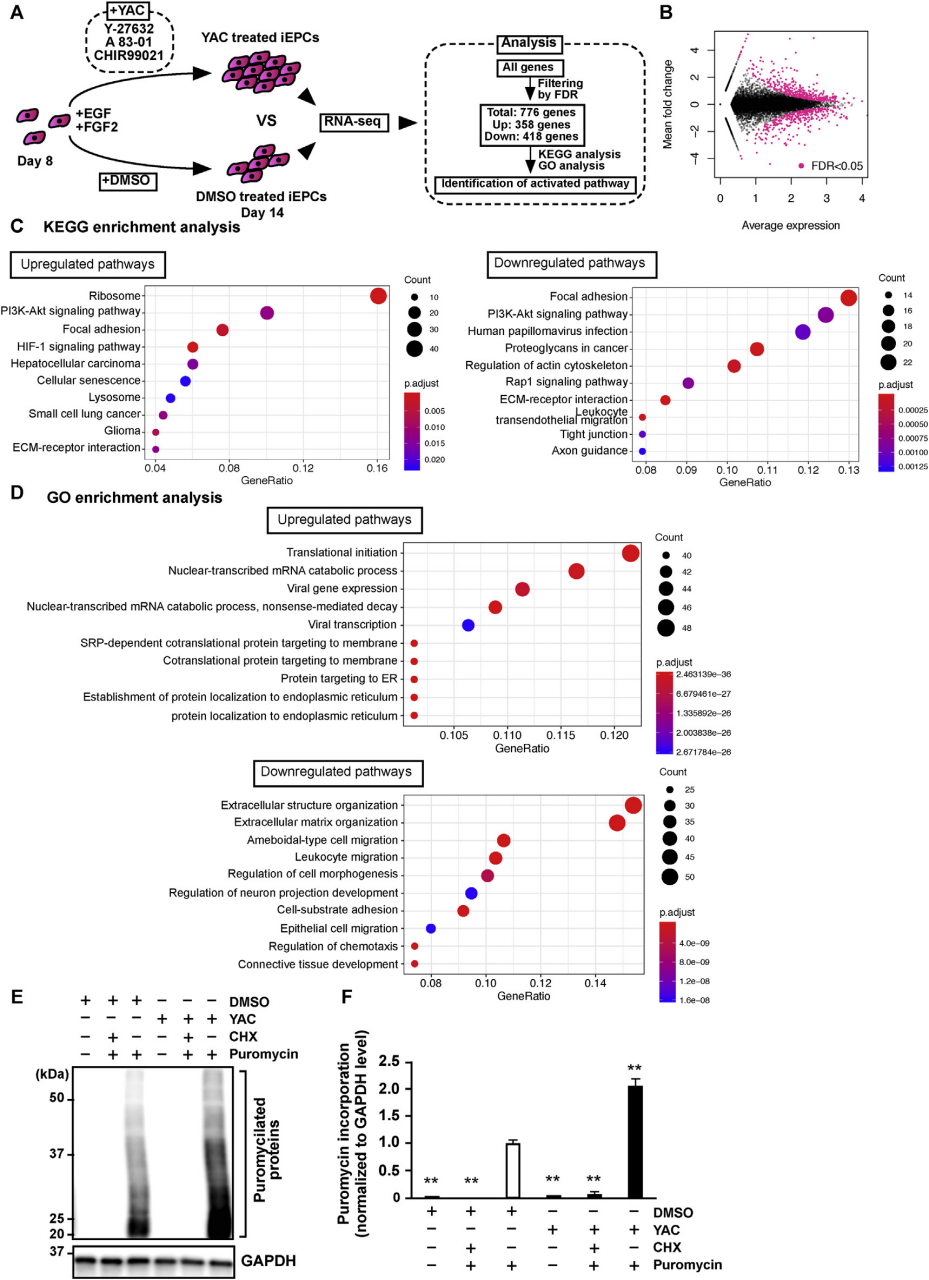
Identification of characteristic biological pathways in YAC-treated iEPCs by RNA-seq. (A) Scheme for the identification of characteristic biological pathways in YAC-treated iEPCs derived from hiPS 610B1 cells by RNA-seq. (B) MA plot of the RNA-seq results. Mean fold change and average expression indicate Log2 (FPKM of YAC-treated iEPCs/FPKM of DMSO-treated iEPCs) and Log10 (FPKM of YAC-treated iEPCs/FPKM of DMSO-treated iEPCs)/2, respectively. Pink dots indicate differentially expressed genes in YAC-treated iEPCs derived from hiPS 610B1 cells. (C) Dotplots of the KEGG enrichment analysis results using ClusterProfiler. (D) Dotplots of the GO enrichment analysis results using ClusterProfiler. (E and F) Puromycin incorporation assay. iEPCs derived from hiPS 610B1 cells were treated with DMSO or YAC from day 11 to day 13. Then, the cells were treated with 1 μg/mL of puromycin for 30 min. Sampling of the cells was conducted on day 13. Cycloheximide (CHX) treatment was performed 10 min before puromycin treatment. Puromycilated proteins were detected by western blot analysis. Puromycin-incorporated protein levels were normalized to GAPDH levels (*n* = 3; ∗∗*p* < 0.01; Games–Howell test; the DMSO (+) and puromycin (+) group vs. others). Uncropped and non-adjusted versions of the immunoblotting data are shown in Fig. S2B.

The activated biological pathways of YAC-treated iEPCs were identified by RNA-seq analysis (Figure 10A). As shown in Tables S1 and S2, 358 genes were upregulated and 418 were downregulated by supplementation with YAC, respectively, as confirmed by a false discovery rate of <0.05 (Figures 10A and 10B). GO and KEGG enrichment analyses were used to identify activated pathways in YAC-treated iEPCs. The results demonstrated the upregulation of genes involved in protein synthesis pathways, such as ribosome, translation initiation, and nuclear-transcribed mRNA catabolic processes (Figures 10C, 10D, S1A, and S1B). Moreover, the results of the puromycin incorporation assay confirmed that YAC significantly activated protein synthesis in iEPCs (Figures 10E and 10F). Meanwhile, genes involved in the regulation of cellular components, such as focal adhesion, regulation of the actin cytoskeleton, tight junctions, and extra structure organization, were downregulated (Figures 10C and 10D). Further validation confirmed that the results of RNA-seq and RT-qPCR analyses were consistent (Fig. S1C).

## 4 Discussion

In this study, a convenient method to supply high-purity and high-quality iEPCs was devised by utilizing the difference in the capacity of cells to adhere to a culture dish. A recent study reported changes in cell properties due to stress induced by the purification process with a cell sorter (Llufrio et al., 2018). Cellular damage caused by the purification processes can be avoided with the use of our proposed purification system. As differentiation progresses, more than two cell types, including iEPCs, emerge, probably because of the heterogeneity of iPSCs (Nguyen et al., 2018) and the presence of cells unresponsive to VEGF among the undifferentiated iPSCs (Ikuno et al., 2017). To confirm the reproducibility of our method, we performed differentiation and purification using three different iPS cell lines and obtained similar outcomes. Therefore, we consider that the reproducibility of this protocol was confirmed. Furthermore, the proposed methods were also successful under the xeno-free condition, suggesting that these protocols can be applied in regenerative medicine. Considering that the proposed purification method does not require an antigen–antibody reaction, the entire process from the establishment of iPSCs to the supply of iEPCs could be accomplished under xeno-free conditions. Human late EPCs (also known as outgrowth ECs) exhibit significantly high expression levels of CD34 and KDR, as compared with HUVECs, as well as strong expression levels of PECAM1 and CDH5 (Cheng et al., 2013; Ormiston et al., 2015; Smadja et al., 2007). iEPCs generated by our method also highly expressed mRNA of CD34 and KDR, as compared with HUVECs. iEPCs also expressed PECAM1 and CDH5. Consistent with previous reports (Harding et al., 2017; Nguyen et al., 2016), iEPCs exhibited a lower expression of vWF than HUVECs, suggesting that the cells were immature. The iEPCs had basic endothelial functions, such as Dil-Ac-LDL uptake and tube formation, similar to those of late EPCs.

The combination of small molecules (YAC) significantly enhanced the proliferation of various cells, including hiPSC-derived posterior gut progenitor cells, rat hepatocytes, and trophoblast stem cells (Okae et al., 2018; Katsuda et al., 2017; Zhang et al., 2018). The results of the present study demonstrated that YAC also stimulates and enhances the proliferative capacity of iEPCs. Surprisingly, iEPCs proliferated by YAC without VEGF, which drives human embryonic stem cell-derived ECs into the venous or arterial phenotype (Sriram et al., 2015), and YAC supplementation during expansion culture maintained the characteristics of EPCs. These results provide new possibilities to robustly expand and supply EPCs, while maintaining the cellular characteristics. Further, YAC has similar effects on HUVECs, suggesting that YAC enhanced the proliferation of broad endothelial lineages for various applications. RNA-seq analysis indicated that YAC stimulates the pathways involved in protein synthesis, including the ribosome pathway and the translation initiation pathway, in iEPCs. Ribosomal proteins, which regulate protein synthesis and basic cellular functions, such as cell proliferation, cell survival, and DNA repair (Xie et al., 2018), were upregulated in YAC-treated iEPCs. YAC also upregulated genes related to the translation initiation pathway, such as eukaryotic initiation factor, which is closely involved in cell survival and proliferation (Shahbazian et al., 2010). Besides, the results of the puromycin incorporation assay indicated that protein synthesis was upregulated in YAC-treated iEPCs. These results suggest that YAC enhances the proliferative capacity of iEPCs through activation of mRNA translation and protein synthesis (Polymenis and Aramayo, 2015).

There were some limitations to this study. First, the proliferative capacity of the iEPCs had decreased on days 30–40 after differentiation even with YAC supplementation of the EPC medium (HE-SFM-based medium). By changing the EPC medium to another EC medium, YAC supplementation would enable greater expansion of iEPCs. A recent study reported the differentiation and expansion of hPSC-derived ECs in three-dimensional (3D) culture (Olmer et al., 2018). However, the use of a 3D culture reduced the expression levels of genes related to cell proliferation (Lin et al., 2018; Zhang et al., 2017b). We speculate that YAC supplementation could also increase cellular proliferation in 3D culture similar to that in two-dimensional culture. By transferring the iEPCs purified by the proposed method to a 3D culture, it might be possible to further expand high-purity cultures. Second, we could not determine which pathway stimulates the protein synthesis pathway. It has been reported that Y-27632 induces the protein synthesis of cardiac muscle cells by inhibiting ET-1 (Kuwahara et al., 1999). In addition, CHIR-99021 has been shown to activate the protein synthesis pathway of hematopoietic stem cells via the activation of mTOR and S6K (Nguyen-McCarty and Klein, 2017). Therefore, these pathways may be involved in the protein synthesis of iEPCs. By elucidating the pathways and mechanisms stimulating the protein synthesis and proliferation of iEPCs by supplementing YAC, it may be possible to increase the amount of iEPCs (and EPCs in the human body) more efficiently by modulating the identified pathway. Third, while we analyzed the effect of freezing–thawing on iEPCs, we did not determine the effect of cryopreservation on iEPCs for long periods of time. Therefore, to determine whether iEPCs can be cryopreserved for a long period of time, further experiments are needed for various applications. Fourth, when we applied iEPCs expanded by YAC to regenerative medicine, the possibility that inhibitors, such as Y-27632, confer tumor potential to the iEPCs cannot be denied. Future studies are warranted to address the harmlessness of cells cultured with these compounds via transplantation experiments in animal models.

## 5 Conclusion

The proposed protocol allows the purification of iEPCs without a complicated selection process and the expansion of culture while maintaining characteristics as EPCs by the addition of three small molecules to the culture medium. These findings should prove useful for the establishment of a stable supply of iEPCs for clinical applications or *in vitro* models.

## Declarations

### Author contribution statement

T. Matsunaga and T. Hashita: Conceived and designed the experiments; Wrote the paper.

H. Aoki: Conceived and designed the experiments; Performed the experiments; Analyzed and interpreted the data; Wrote the paper.

M. Yamashita: Performed the experiments; Analyzed and interpreted the data; Wrote the paper.

K. Ogami: Performed the experiments; Analyzed and interpreted the data; Contributed reagents, materials, analysis tools or data; Wrote the paper.

S. Hoshino: Contributed reagents, materials, analysis tools or data; Wrote the paper.

T. Iwao: Analyzed and interpreted the data; Wrote the paper.

### Funding statement

This work was supported by AMED [Grant Number JP19be0304322] and JSPS KAKENHI [Grant Number JP17K08422].

### Competing interest statement

The authors declare no conflict of interest.

### Additional information

Data associated with this study has been deposited at NCBI Gene Expression Omnibus under the accession number GSE138225.

Supplementary content related to this article has been published online at https://doi.org/10.1016/j.heliyon.2020.e03493.

## Appendix A Supplementary data

iEPCs Heliyon supplementaly file iEPCs Heliyon supplementaly file
