# Supplementary material for: Efficient differentiation and purification of human induced pluripotent stem cell-derived endothelial progenitor cells and expansion with the use of inhibitors of ROCK, TGF-β, and GSK3β
Source: Heliyon. 2020 Mar 3;6(3):e03493. doi: 10.1016/j.heliyon.2020.e03493 (PMC7056658; doi:10.1016/j.heliyon.2020.e03493)
Supplement: iEPCs Heliyon supplementaly file [file mmc1.pdf]

Supplementary figures and tables

Efficient differentiation and purification of human induced pluripotent stem cell-derived endothelial progenitor cells and expansion with the use of inhibitors of ROCK, TGF-β, and GSK3β

Hiromasa Aoki, Misaki Yamashita, Tadahiro Hashita, Koichi Ogami, Shinichi Hoshino, Takahiro Iwao, Tamihide Matsunaga

Components
Two Supplemental Figures and two Supplemental Tables

Supplementary Figures

Figure S1

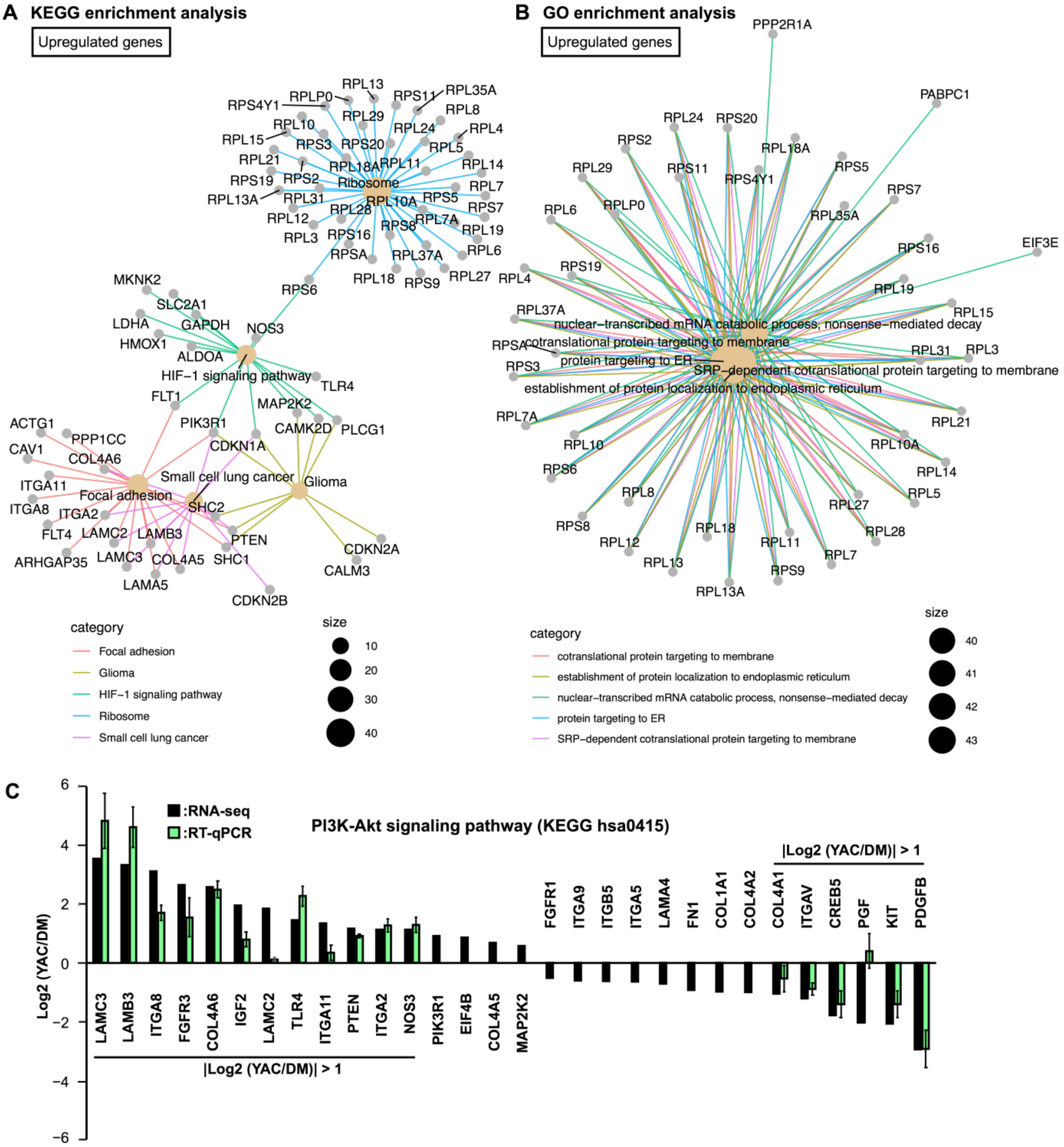

Fig. S1. Gene expression analysis of YAC-treated 610B1-derived iEPCs by RNA-seq analysis.
(A) Cnetplot for the results of KEGG enrichment analysis of upregulated genes using ClusterProfiler.
(B) Cnetplot for the results of GO enrichment analysis of upregulated genes using ClusterProfiler.
(C) Validation of the results of RNA-seq (KEGG hsa0415). Relative expression levels of selected genes determined by FDR below 0.05 and 1 < |Log2 [FPKM of YAC group (YAC)/FPKM of DMSO group (DM)]| were analyzed by RT-qPCR. Data are presented as mean ± SD (*n* = 3).

Figure S2

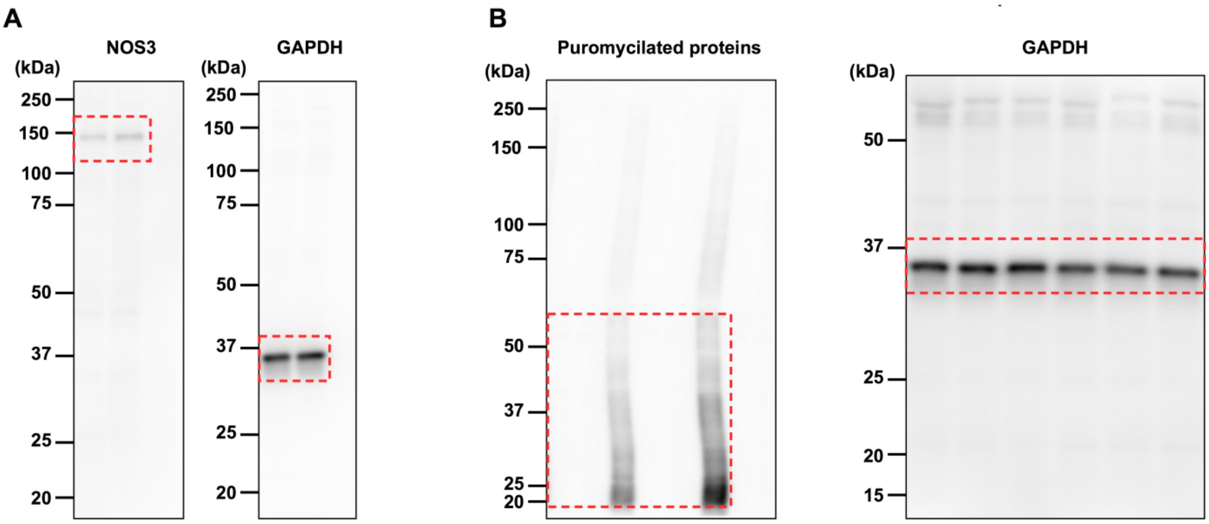

Fig. S2. Uncropped and non-adjusted versions of the immunoblot data.
(A) Uncropped and non-adjusted versions of the immunoblot data of Fig. 8C are shown.
(B) Uncropped and non-adjusted versions of the immunoblot data of Fig. 10E are shown.

Table S1. Upregulated genes in YAC-treated iEPCs

Gene_id	hgnc_symbol	log2.Fold_change	*p* value	q value
ENSG00000133636	NTS	6.6759	0.0010572	0.031766
ENSG00000125798	FOXA2	6.6702	0.00016033	0.0060904
ENSG00000167434	CA4	6.2695	4.05E-08	3.06E-06
ENSG00000171303	KCNK3	5.7938	0.00056178	0.01858
ENSG00000116132	PRRX1	5.7055	3.22E-07	2.19E-05
ENSG00000138061	CYP1B1	5.2471	8.35E-10	7.91E-08
ENSG00000154856	APCDD1	5.1877	1.10E-05	0.00055413
ENSG00000140465	CYP1A1	4.9331	2.70E-114	4.24E-111
ENSG00000100985	MMP9	4.9057	0	0
ENSG00000068615	REEP1	4.6994	2.39E-05	0.0011295
ENSG00000123689	G0S2	4.5639	0.00014327	0.0055144
ENSG00000135144	DTX1	3.966	8.98E-06	0.00046145
ENSG00000135218	CD36	3.9144	0.00076655	0.024085
ENSG00000117394	SLC2A1	3.8493	1.60E-104	2.19E-101
ENSG00000135838	NPL	3.8222	8.03E-11	8.49E-09
ENSG00000050555	LAMC3	3.5433	3.30E-09	2.87E-07
ENSG00000137491	SLCO2B1	3.4961	1.18E-05	0.00059111
ENSG00000063438	AHRR	3.4354	1.59E-10	1.63E-08
ENSG00000092068	SLC7A8	3.4155	2.38E-43	1.54E-40
ENSG00000136011	STAB2	3.3581	1.19E-05	0.00059716
ENSG00000196878	LAMB3	3.3205	4.15E-17	7.74E-15
ENSG00000169783	LINGO1	3.2806	0.00040783	0.014193
ENSG00000161653	NAGS	3.1822	0.0014393	0.041819
ENSG00000168646	AXIN2	3.1154	5.02E-06	0.00027446
ENSG00000077943	ITGA8	3.1136	5.11E-17	9.44E-15
ENSG00000133083	DCLK1	3.1076	0.0001327	0.0051657
ENSG00000197467	COL13A1	3.0106	8.27E-05	0.003419
ENSG00000099998	GGT5	2.8108	5.62E-07	3.67E-05
ENSG00000115461	IGFBP5	2.7476	2.50E-30	9.84E-28
ENSG00000102575	ACP5	2.7373	6.33E-23	1.64E-20
ENSG00000167106	FAM102A	2.7067	1.02E-38	5.45E-36
ENSG00000167779	IGFBP6	2.6539	3.87E-05	0.0017573
ENSG00000068078	FGFR3	2.6438	0.00060347	0.019692
ENSG00000004799	PDK4	2.6217	7.82E-13	1.08E-10
ENSG00000187773	FAM69C	2.5757	0.00083486	0.025789

ENSG00000197565	COL4A6	2.5735	2.33E-11	2.70E-09
ENSG00000185551	NR2F2	2.5681	7.98E-44	5.32E-41
ENSG00000197355	UAP1L1	2.4946	4.90E-20	1.13E-17
ENSG00000175445	LPL	2.4918	1.52E-48	1.19E-45
ENSG00000090776	EFNB1	2.4499	1.32E-09	1.21E-07
ENSG00000136160	EDNRB	2.427	3.99E-05	0.0018019
ENSG00000061337	LZTS1	2.4246	0.0015382	0.044281
ENSG00000270547	LINC01235	2.4163	2.22E-14	3.51E-12
ENSG00000159307	SCUBE1	2.4119	0.00078931	0.024624
ENSG00000111181	SLC6A12	2.2842	0.0010375	0.031215
ENSG00000111885	MAN1A1	2.1931	1.01E-12	1.37E-10
ENSG00000130600	H19	2.1845	1.16E-135	3.18E-132
ENSG00000148908	RGS10	2.154	5.90E-09	4.99E-07
ENSG00000168497	CAVIN2	2.1129	2.19E-09	1.92E-07
ENSG00000137965	IFI44	2.0746	0.00063236	0.020544
ENSG00000144681	STAC	2.0099	0.0016685	0.047411
ENSG00000125845	BMP2	1.9834	1.19E-06	7.26E-05
ENSG00000204634	TBC1D8	1.9705	1.22E-11	1.47E-09
ENSG00000134569	LRP4	1.9549	2.09E-05	0.0010017
ENSG00000135549	PKIB	1.9491	0.00040131	0.01401
ENSG00000167244	IGF2	1.9383	8.81E-23	2.25E-20
ENSG00000177721	ANXA2R	1.9316	0.00051649	0.017297
ENSG00000181804	SLC9A9	1.898	0.00031327	0.011131
ENSG00000170425	ADORA2B	1.8904	7.58E-06	0.00039801
ENSG00000181019	NQO1	1.8798	2.28E-21	5.57E-19
ENSG00000130830	MPP1	1.8752	1.80E-14	2.89E-12
ENSG00000120885	CLU	1.8421	1.60E-08	1.27E-06
ENSG00000058085	LAMC2	1.8347	0.00034148	0.012075
ENSG00000118777	ABCG2	1.7644	5.03E-05	0.0021955
ENSG00000204103	MAFB	1.7472	4.72E-20	1.10E-17
ENSG00000071575	TRIB2	1.7243	0.00013742	0.0053305
ENSG00000176641	RNF152	1.7238	3.56E-06	0.00020106
ENSG00000140450	ARRDC4	1.7056	1.13E-07	8.14E-06
ENSG00000124440	HIF3A	1.6909	1.07E-09	1.00E-07
ENSG00000090530	P3H2	1.6785	0.00021245	0.0078929
ENSG00000165655	ZNF503	1.6775	1.18E-18	2.50E-16
ENSG00000151012	SLC7A11	1.6676	4.88E-08	3.65E-06

ENSG00000147872	PLIN2	1.6574	1.34E-16	2.41E-14
ENSG00000164543	STK17A	1.6558	0.00012556	0.00494
ENSG00000130513	GDF15	1.6386	2.98E-20	7.19E-18
ENSG00000163686	ABHD6	1.6347	6.01E-06	0.0003223
ENSG00000135709	KIAA0513	1.6048	0.0016423	0.046909
ENSG00000179630	LACC1	1.5825	9.93E-05	0.0040234
ENSG00000165379	LRFN5	1.5659	0.0015282	0.044051
ENSG00000099875	MKNK2	1.5642	5.54E-69	5.80E-66
ENSG00000163132	MSX1	1.5504	1.80E-09	1.60E-07
ENSG00000100504	PYGL	1.5447	4.08E-07	2.73E-05
ENSG00000186642	PDE2A	1.5423	5.15E-10	5.06E-08
ENSG00000124191	TOX2	1.5253	5.87E-10	5.69E-08
ENSG00000166450	PRTG	1.5245	9.21E-06	0.00047216
ENSG00000138119	MYOF	1.5223	1.31E-12	1.74E-10
ENSG00000137309	HMGA1	1.4878	4.43E-51	3.89E-48
ENSG00000163762	TM4SF18	1.4836	2.93E-05	0.001366
ENSG00000101577	LPIN2	1.4809	0.00030065	0.010735
ENSG00000196141	SPATS2L	1.4789	5.06E-16	8.83E-14
ENSG00000013016	EHD3	1.4641	5.13E-11	5.53E-09
ENSG00000136869	TLR4	1.4432	6.58E-06	0.00034983
ENSG00000159167	STC1	1.4278	4.12E-20	9.75E-18
ENSG00000175745	NR2F1	1.4221	1.33E-09	1.21E-07
ENSG00000138166	DUSP5	1.4215	4.29E-08	3.23E-06
ENSG00000087303	NID2	1.3953	8.58E-13	1.17E-10
ENSG00000103449	SALL1	1.3951	0.0014194	0.041315
ENSG00000143603	KCNN3	1.3855	0.00133	0.039212
ENSG00000100285	NEFH	1.3817	8.39E-05	0.0034631
ENSG00000109099	PMP22	1.3769	8.04E-13	1.11E-10
ENSG00000088367	EPB41L1	1.3751	3.69E-08	2.80E-06
ENSG00000136960	ENPP2	1.363	2.14E-06	0.00012603
ENSG00000188848	BEND4	1.3473	1.24E-08	1.00E-06
ENSG00000119138	KLF9	1.346	0.0012526	0.03708
ENSG00000137809	ITGA11	1.34	0.00064149	0.020779
ENSG00000135678	CPM	1.3396	2.53E-18	5.11E-16
ENSG00000185339	TCN2	1.3076	3.04E-06	0.00017347
ENSG00000028137	TNFRSF1B	1.2916	1.59E-29	5.82E-27
ENSG00000178718	RPP25	1.2828	3.00E-08	2.33E-06

ENSG00000175899	A2M	1.277	5.66E-06	0.00030464
ENSG00000153208	MERTK	1.2763	0.00046276	0.01578
ENSG00000173559	NABP1	1.2647	7.32E-07	4.65E-05
ENSG00000137449	CPEB2	1.2608	2.15E-09	1.89E-07
ENSG00000169855	ROBO1	1.2562	4.12E-16	7.25E-14
ENSG00000137266	SLC22A23	1.2424	7.09E-19	1.54E-16
ENSG00000106266	SNX8	1.2397	6.87E-07	4.42E-05
ENSG00000042493	CAPG	1.215	0.00065245	0.021041
ENSG00000139977	NAA30	1.2063	4.49E-12	5.64E-10
ENSG00000138162	TACC2	1.1974	1.78E-09	1.59E-07
ENSG00000250722	SELENOP	1.1844	1.06E-07	7.66E-06
ENSG00000164904	ALDH7A1	1.1731	2.54E-15	4.34E-13
ENSG00000102032	RENBP	1.1723	6.12E-06	0.00032763
ENSG00000171862	PTEN	1.158	3.94E-06	0.00022173
ENSG00000141504	SAT2	1.1526	0.00012466	0.0049137
ENSG00000184005	ST6GALNAC3	1.152	0.00064528	0.020871
ENSG00000164403	SHROOM1	1.15	0.0015479	0.044445
ENSG00000127824	TUBA4A	1.1493	0.00070314	0.022316
ENSG00000147883	CDKN2B	1.1437	7.16E-05	0.003011
ENSG00000197852	INKA2	1.14	0.00070641	0.022387
ENSG00000076944	STXBP2	1.1391	0.0016404	0.046909
ENSG00000164171	ITGA2	1.1257	1.39E-09	1.26E-07
ENSG00000164867	NOS3	1.1244	3.49E-29	1.26E-26
ENSG00000213699	SLC35F6	1.1128	0.00046862	0.01593
ENSG00000173210	ABLIM3	1.1116	0.00049922	0.016814
ENSG00000069702	TGFBR3	1.1032	3.66E-12	4.68E-10
ENSG00000166780	C16orf45	1.089	9.49E-06	0.00048333
ENSG00000120645	IQSEC3	1.0841	0.00026612	0.0096586
ENSG00000135362	PRR5L	1.0833	3.23E-06	0.00018371
ENSG00000120875	DUSP4	1.0723	2.51E-14	3.94E-12
ENSG00000145349	CAMK2D	1.0656	0.0014201	0.041315
ENSG00000149948	HMGA2	1.0636	1.07E-06	6.57E-05
ENSG00000118640	VAMP8	1.0615	1.61E-06	9.61E-05
ENSG00000168003	SLC3A2	1.0573	2.55E-12	3.30E-10
ENSG00000104763	ASAH1	1.0394	4.89E-11	5.32E-09
ENSG00000205302	SNX2	1.0268	5.46E-06	0.00029566
ENSG00000064989	CALCRL	1.0221	2.96E-07	2.02E-05

ENSG00000133800	LYVE1	1.0164	5.16E-30	1.95E-27
ENSG00000108175	ZMIZ1	1.015	2.19E-18	4.46E-16
ENSG00000099864	PALM	1.0131	3.33E-14	5.20E-12
ENSG00000184602	SNN	1.006	4.15E-13	5.92E-11
ENSG00000160789	LMNA	1.0044	4.57E-26	1.36E-23
ENSG00000186815	TPCN1	1.0038	1.27E-06	7.71E-05
ENSG00000189060	H1F0	0.9945	8.47E-07	5.33E-05
ENSG00000135111	TBX3	0.99322	0.00045819	0.015648
ENSG00000130066	SAT1	0.98513	4.51E-43	2.68E-40
ENSG00000196329	GIMAP5	0.97694	7.14E-05	0.0030086
ENSG00000113328	CCNG1	0.97592	1.73E-06	0.00010303
ENSG00000163931	TKT	0.97539	1.19E-18	2.50E-16
ENSG00000174718	KIAA1551	0.97204	9.01E-10	8.47E-08
ENSG00000121060	TRIM25	0.97123	0.00067142	0.021495
ENSG00000147416	ATP6V1B2	0.96173	7.76E-09	6.47E-07
ENSG00000063660	GPC1	0.95466	7.47E-06	0.00039296
ENSG00000205336	ADGRG1	0.95424	6.63E-08	4.91E-06
ENSG00000168010	ATG16L2	0.94775	2.08E-10	2.10E-08
ENSG00000213203	GIMAP1	0.94559	8.89E-05	0.0036351
ENSG00000157617	C2CD2	0.94275	0.0014914	0.043162
ENSG00000147889	CDKN2A	0.94222	7.40E-05	0.0030871
ENSG00000100292	HMOX1	0.93996	7.48E-10	7.15E-08
ENSG00000177000	MTHFR	0.93698	0.00082166	0.025489
ENSG00000107521	HPS1	0.93509	7.22E-08	5.31E-06
ENSG00000145675	PIK3R1	0.91603	0.00029393	0.010546
ENSG00000182287	AP1S2	0.91322	8.50E-06	0.00043899
ENSG00000087076	HSD17B14	0.91263	0.00039339	0.013756
ENSG00000115866	DARS	0.91045	0.00052928	0.017638
ENSG00000182022	CHST15	0.90633	9.69E-08	7.04E-06
ENSG00000181649	PHLDA2	0.90479	3.80E-05	0.0017295
ENSG00000178573	MAF	0.89828	7.47E-07	4.74E-05
ENSG00000100060	MFNG	0.8964	5.38E-10	5.24E-08
ENSG00000113552	GNPDA1	0.87396	0.00077213	0.024226
ENSG00000164713	BRI3	0.8737	2.97E-10	2.97E-08
ENSG00000013364	MVP	0.87259	0.00055892	0.018513
ENSG00000175832	ETV4	0.86674	0.00069578	0.022146
ENSG00000122359	ANXA11	0.86445	5.76E-11	6.15E-09

ENSG00000104518	GSDMD	0.86289	1.36E-06	8.25E-05
ENSG00000063046	EIF4B	0.85614	2.25E-17	4.27E-15
ENSG00000116396	KCNC4	0.8509	1.28E-05	0.00063299
ENSG00000136840	ST6GALNAC4	0.84974	9.47E-06	0.00048333
ENSG00000113389	NPR3	0.84509	7.01E-10	6.74E-08
ENSG00000185650	ZFP36L1	0.84498	1.81E-11	2.15E-09
ENSG00000110237	ARHGEF17	0.83692	7.79E-31	3.17E-28
ENSG00000006831	ADIPOR2	0.81917	1.71E-07	1.20E-05
ENSG00000214063	TSPAN4	0.8168	0.00015898	0.0060497
ENSG00000196562	SULF2	0.81622	1.21E-25	3.55E-23
ENSG00000126261	UBA2	0.81606	0.00057831	0.018984
ENSG00000118257	NRP2	0.80535	1.38E-12	1.82E-10
ENSG00000167658	EEF2	0.80281	4.96E-128	1.21E-124
ENSG00000118816	CCNI	0.79956	4.05E-05	0.0018157
ENSG00000162783	IER5	0.79186	0.00050687	0.017046
ENSG00000103187	COTL1	0.78964	7.18E-15	1.19E-12
ENSG00000124299	PEPD	0.78588	0.00020407	0.0076073
ENSG00000141985	SH3GL1	0.78344	2.70E-10	2.72E-08
ENSG00000198959	TGM2	0.77721	8.03E-49	6.54E-46
ENSG00000112031	MTRF1L	0.77609	2.91E-06	0.00016753
ENSG00000119655	NPC2	0.77573	0.00052639	0.017568
ENSG00000153207	AHCTF1	0.77485	0.0016493	0.04705
ENSG00000233016	SNHG7	0.7589	0.0007369	0.023253
ENSG00000182378	PLCXD1	0.75818	0.0014896	0.043162
ENSG00000168890	TMEM150A	0.75109	0.00078375	0.024485
ENSG00000103018	CYB5B	0.75021	1.69E-07	1.19E-05
ENSG00000234741	GAS5	0.74631	0.00081948	0.025457
ENSG00000163399	ATP1A1	0.74457	8.49E-20	1.92E-17
ENSG00000169291	SHE	0.74194	2.74E-08	2.14E-06
ENSG00000185909	KLHDC8B	0.73961	8.50E-07	5.33E-05
ENSG00000125651	GTF2F1	0.73613	3.26E-05	0.0015106
ENSG00000176788	BASP1	0.73262	4.87E-06	0.00026787
ENSG00000138434	ITPRID2	0.72891	1.06E-11	1.28E-09
ENSG00000144711	IQSEC1	0.72402	1.35E-07	9.62E-06
ENSG00000042753	AP2S1	0.71821	5.36E-11	5.75E-09
ENSG00000169100	SLC25A6	0.71382	7.52E-22	1.86E-19
ENSG00000204386	NEU1	0.71286	3.64E-05	0.0016692

ENSG00000138095	LRPPRC	0.71278	0.00014288	0.0055133
ENSG00000132535	DLG4	0.7107	0.00016383	0.0062019
ENSG00000090372	STRN4	0.70987	1.62E-14	2.66E-12
ENSG00000154864	PIEZO2	0.70744	5.33E-09	4.53E-07
ENSG00000167693	NXN	0.70638	0.0011586	0.034482
ENSG00000118960	HS1BP3	0.70263	0.0016989	0.048143
ENSG00000184465	WDR27	0.70046	6.87E-05	0.0029115
ENSG00000007047	MARK4	0.69692	1.71E-08	1.36E-06
ENSG00000085117	CD82	0.69048	3.03E-06	0.00017345
ENSG00000142910	TINAGL1	0.68559	0.00082775	0.025637
ENSG00000111716	LDHB	0.68447	1.70E-13	2.49E-11
ENSG00000188153	COL4A5	0.68245	0.00015326	0.005852
ENSG00000136720	HS6ST1	0.68084	1.26E-09	1.17E-07
ENSG00000148180	GSN	0.67283	1.17E-07	8.39E-06
ENSG00000134333	LDHA	0.66894	0.0001259	0.0049445
ENSG00000115486	GGCX	0.66628	0.00066365	0.021309
ENSG00000181163	NPM1	0.66503	1.14E-11	1.37E-09
ENSG00000105483	CARD8	0.66354	4.08E-05	0.0018222
ENSG00000137507	LRRC32	0.66289	4.14E-06	0.00023083
ENSG00000005022	SLC25A5	0.6619	3.67E-05	0.0016759
ENSG00000152556	PFKM	0.66155	0.0002267	0.0083516
ENSG00000196961	AP2A1	0.6608	8.57E-10	8.09E-08
ENSG00000160014	CALM3	0.65844	4.43E-12	5.60E-10
ENSG00000167323	STIM1	0.65661	0.00017991	0.0067872
ENSG00000205726	ITSN1	0.65428	0.00045176	0.015503
ENSG00000105373	NOP53	0.65175	3.22E-08	2.48E-06
ENSG00000197958	RPL12	0.64966	4.76E-09	4.06E-07
ENSG00000024422	EHD2	0.64955	6.87E-15	1.15E-12
ENSG00000163171	CDC42EP3	0.64644	5.67E-06	0.00030464
ENSG00000112335	SNX3	0.6419	5.65E-08	4.21E-06
ENSG00000197081	IGF2R	0.64142	1.79E-14	2.89E-12
ENSG00000152767	FARP1	0.63691	4.54E-05	0.0020004
ENSG00000115902	SLC1A4	0.63446	6.59E-07	4.28E-05
ENSG00000130741	EIF2S3	0.63402	0.00028008	0.010099
ENSG00000105220	GPI	0.63082	3.38E-07	2.29E-05
ENSG00000245532	NEAT1	0.62822	6.56E-09	5.51E-07
ENSG00000240342	RPS2P5	0.62684	4.02E-05	0.0018084

ENSG00000137509	PRCP	0.62674	7.07E-08	5.22E-06
ENSG00000161011	SQSTM1	0.62081	9.82E-06	0.00049869
ENSG00000128641	MYO1B	0.61955	0.0015578	0.04467
ENSG00000100280	AP1B1	0.61813	4.92E-22	1.23E-19
ENSG00000079819	EPB41L2	0.61424	4.60E-07	3.04E-05
ENSG00000196839	ADA	0.61258	0.00069176	0.022082
ENSG00000104408	EIF3E	0.60482	6.67E-05	0.0028371
ENSG00000182871	COL18A1	0.60393	8.47E-34	4.05E-31
ENSG00000143612	C1orf43	0.6006	0.00088413	0.027045
ENSG00000116133	DHCR24	0.59705	1.10E-10	1.14E-08
ENSG00000073578	SDHA	0.59279	0.00074898	0.023566
ENSG00000107581	EIF3A	0.59006	4.09E-09	3.52E-07
ENSG00000214655	ZSWIM8	0.58838	7.33E-05	0.0030698
ENSG00000010327	STAB1	0.5882	1.50E-28	5.07E-26
ENSG00000059804	SLC2A3	0.58743	2.90E-05	0.0013538
ENSG00000182511	FES	0.58621	0.0016665	0.047411
ENSG00000100353	EIF3D	0.5836	0.000127	0.0049788
ENSG00000070756	PABPC1	0.58137	7.04E-18	1.37E-15
ENSG00000126934	MAP2K2	0.57491	5.17E-06	0.00028201
ENSG00000186298	PPP1CC	0.57215	0.0017503	0.049355
ENSG00000140988	RPS2	0.56963	7.62E-33	3.49E-30
ENSG00000183580	FBXL7	0.56685	0.0013649	0.039973
ENSG00000129946	SHC2	0.56576	0.00013147	0.0051267
ENSG00000144381	HSPD1	0.56327	3.95E-05	0.0017861
ENSG00000155463	OXA1L	0.56317	0.00088189	0.027014
ENSG00000100600	LGMN	0.56207	0.00057265	0.018826
ENSG00000116016	EPAS1	0.56124	1.11E-18	2.36E-16
ENSG00000129824	RPS4Y1	0.56015	0.0013598	0.039875
ENSG00000129538	RNASE1	0.55951	1.71E-05	0.00083217
ENSG00000110841	PPFIBP1	0.55833	0.00010977	0.0044137
ENSG00000169710	FASN	0.55592	2.03E-28	6.76E-26
ENSG00000107404	DVL1	0.54665	0.00079333	0.024715
ENSG00000169908	TM4SF1	0.54206	0.00013475	0.0052361
ENSG00000137575	SDCBP	0.53945	8.94E-05	0.0036464
ENSG00000148337	CIZ1	0.5376	0.001353	0.039731
ENSG00000064393	HIPK2	0.53385	5.50E-07	3.60E-05
ENSG00000233276	GPX1	0.53352	1.01E-13	1.52E-11

ENSG00000203485	INF2	0.52473	7.19E-07	4.58E-05
ENSG00000135636	DYSF	0.52383	2.23E-11	2.59E-09
ENSG00000078902	TOLLIP	0.51956	0.001348	0.039635
ENSG00000183688	RFLNB	0.51944	0.00045024	0.015497
ENSG00000142541	RPL13A	0.51729	3.66E-23	9.58E-21
ENSG00000064601	CTSA	0.51692	0.00020025	0.0074905
ENSG00000100316	RPL3	0.51667	5.89E-24	1.60E-21
ENSG00000160691	SHC1	0.51338	2.54E-15	4.34E-13
ENSG00000135677	GNS	0.51272	9.57E-12	1.17E-09
ENSG00000184640	SEPT9	0.51161	1.63E-10	1.66E-08
ENSG00000183963	SMTN	0.50968	1.84E-18	3.77E-16
ENSG00000105193	RPS16	0.50675	2.77E-09	2.42E-07
ENSG00000182718	ANXA2	0.50525	3.21E-20	7.69E-18
ENSG00000107816	LZTS2	0.50403	0.00036459	0.01285
ENSG00000122705	CLTA	0.50329	0.00044725	0.015418
ENSG00000122406	RPL5	0.50302	7.72E-10	7.35E-08
ENSG00000108219	TSPAN14	0.50034	0.000208	0.0077407
ENSG00000089351	GRAMD1A	0.49899	7.19E-05	0.0030197
ENSG00000066735	KIF26A	0.49855	3.46E-05	0.0015897
ENSG00000075785	RAB7A	0.48986	1.45E-05	0.00071535
ENSG00000105974	CAV1	0.48861	0.00047561	0.016118
ENSG00000152234	ATP5F1A	0.48254	8.28E-06	0.00043067
ENSG00000178719	GRINA	0.48242	0.00070306	0.022316
ENSG00000169992	NLGN2	0.4767	0.00036853	0.012927
ENSG00000204628	RACK1	0.47569	3.02E-11	3.42E-09
ENSG00000111252	SH2B3	0.47526	4.53E-12	5.66E-10
ENSG00000083845	RPS5	0.47499	1.69E-07	1.19E-05
ENSG00000184916	JAG2	0.47407	0.00012849	0.0050284
ENSG00000173166	RAPH1	0.47379	0.00024766	0.0090635
ENSG00000215021	PHB2	0.47277	0.00049779	0.016792
ENSG00000137154	RPS6	0.47256	4.34E-11	4.75E-09
ENSG00000168028	RPSA	0.47245	1.28E-10	1.32E-08
ENSG00000129657	SEC14L1	0.46911	1.26E-12	1.68E-10
ENSG00000133612	AGAP3	0.46535	5.48E-05	0.002367
ENSG00000110955	ATP5F1B	0.46374	2.23E-08	1.75E-06
ENSG00000160007	ARHGAP35	0.46166	0.0008055	0.025058
ENSG00000107372	ZFAND5	0.46085	0.0013464	0.039635

ENSG00000170889	RPS9	0.45711	3.75E-11	4.15E-09
ENSG00000149273	RPS3	0.45632	1.30E-17	2.51E-15
ENSG00000063245	EPN1	0.45612	9.23E-09	7.61E-07
ENSG00000148303	RPL7A	0.45526	2.91E-11	3.32E-09
ENSG00000063177	RPL18	0.45466	8.63E-07	5.39E-05
ENSG00000156508	EEF1A1	0.45268	8.10E-64	8.09E-61
ENSG00000087086	FTL	0.4508	4.64E-09	3.97E-07
ENSG00000026025	VIM	0.44945	1.58E-09	1.42E-07
ENSG00000142192	APP	0.44877	6.47E-33	3.03E-30
ENSG00000197157	SND1	0.44827	0.00015425	0.0058796
ENSG00000147604	RPL7	0.44519	7.26E-09	6.07E-07
ENSG00000111640	GAPDH	0.44516	6.22E-20	1.43E-17
ENSG00000142676	RPL11	0.44232	2.48E-05	0.0011688
ENSG00000168488	ATXN2L	0.44051	0.00022855	0.0084057
ENSG00000108828	VAT1	0.43572	8.61E-09	7.12E-07
ENSG00000108107	RPL28	0.43541	4.04E-09	3.48E-07
ENSG00000105568	PPP2R1A	0.43267	0.00058404	0.019144
ENSG00000065000	AP3D1	0.43264	4.01E-05	0.0018072
ENSG00000131469	RPL27	0.4286	8.56E-06	0.00044079
ENSG00000188846	RPL14	0.42716	7.09E-06	0.00037562
ENSG00000101384	JAG1	0.42521	0.0017544	0.049405
ENSG00000198755	RPL10A	0.42047	1.19E-05	0.00059716
ENSG00000145741	BTF3	0.41935	0.00097746	0.029745
ENSG00000257093	KIAA1147	0.41727	5.04E-05	0.0021955
ENSG00000251322	SHANK3	0.41338	6.45E-29	2.29E-26
ENSG00000130702	LAMA5	0.41221	1.63E-27	5.04E-25
ENSG00000161203	AP2M1	0.40983	2.49E-07	1.72E-05
ENSG00000187109	NAP1L1	0.40808	0.00022165	0.0081793
ENSG00000088247	KHSRP	0.40212	7.67E-06	0.0004015
ENSG00000108298	RPL19	0.40201	4.87E-05	0.0021301
ENSG00000161016	RPL8	0.39991	6.11E-12	7.59E-10
ENSG00000171863	RPS7	0.3978	0.0007786	0.024359
ENSG00000115677	HDLBP	0.39559	3.04E-06	0.00017345
ENSG00000174444	RPL4	0.39281	5.38E-10	5.24E-08
ENSG00000114391	RPL24	0.38506	0.00054668	0.018163
ENSG00000196611	MMP1	0.38444	1.95E-05	0.00094061
ENSG00000009307	CSDE1	0.38044	0.00048276	0.016335

ENSG00000075415	SLC25A3	0.37968	0.00012228	0.0048283
ENSG00000182899	RPL35A	0.37546	0.00045182	0.015503
ENSG00000008988	RPS20	0.37506	0.00032086	0.011382
ENSG00000124181	PLCG1	0.37419	0.00027605	0.0099858
ENSG00000138834	MAPK8IP3	0.3737	1.06E-05	0.00053593
ENSG00000067225	PKM	0.37152	1.51E-07	1.07E-05
ENSG00000162244	RPL29	0.36957	1.48E-05	0.00072607
ENSG00000022567	SLC45A4	0.36773	4.20E-06	0.00023354
ENSG00000124762	CDKN1A	0.36607	7.72E-06	0.00040356
ENSG00000089157	RPLP0	0.36591	3.55E-13	5.13E-11
ENSG00000104870	FCGRT	0.36354	0.00029947	0.01071
ENSG00000105701	FKBP8	0.35995	0.00021594	0.008009
ENSG00000142937	RPS8	0.35862	4.29E-05	0.0018999
ENSG00000196531	NACA	0.35853	0.0015773	0.045169
ENSG00000122026	RPL21	0.35685	0.0008721	0.026789
ENSG00000137409	MTCH1	0.35511	0.0004245	0.01468
ENSG00000198840	MT-ND3	0.35021	0.0006194	0.020152
ENSG00000167526	RPL13	0.33648	1.10E-06	6.74E-05
ENSG00000106682	EIF4H	0.33138	0.0013815	0.040352
ENSG00000089009	RPL6	0.33122	9.98E-07	6.17E-05
ENSG00000105401	CDC37	0.31564	0.0010734	0.03217
ENSG00000123143	PKN1	0.31532	4.10E-05	0.0018246
ENSG00000141524	TMC6	0.31399	0.0011093	0.033149
ENSG00000163513	TGFBR2	0.31278	5.62E-06	0.0003035
ENSG00000065978	YBX1	0.31257	1.43E-06	8.59E-05
ENSG00000136830	FAM129B	0.30988	6.90E-05	0.0029171
ENSG00000105640	RPL18A	0.30852	2.76E-05	0.0012975
ENSG00000142534	RPS11	0.30761	1.27E-05	0.00063056
ENSG00000071082	RPL31	0.3074	0.0015083	0.043592
ENSG00000184113	CLDN5	0.30071	8.01E-07	5.05E-05
ENSG00000135486	HNRNPA1	0.29433	0.00014485	0.0055599
ENSG00000174748	RPL15	0.28332	0.00085718	0.026404
ENSG00000147403	RPL10	0.28298	9.30E-05	0.0037874
ENSG00000102755	FLT1	0.27765	1.37E-06	8.27E-05
ENSG00000071242	RPS6KA2	0.27692	0.00066721	0.021391
ENSG00000197756	RPL37A	0.27633	0.0017008	0.048143
ENSG00000149925	ALDOA	0.27259	0.00019099	0.0071562

ENSG00000198886	MT-ND4	0.27211	4.13E-07	2.75E-05
ENSG00000096384	HSP90AB1	0.2596	5.33E-06	0.00029008
ENSG00000105372	RPS19	0.24538	0.0015461	0.044445
ENSG00000129968	ABHD17A	0.24283	0.00066152	0.021271
ENSG00000198804	MT-CO1	0.2353	5.24E-30	1.95E-27
ENSG00000172889	EGFL7	0.22565	4.53E-06	0.00025036
ENSG00000037280	FLT4	0.22539	1.63E-05	0.00079682
ENSG00000198899	MT-ATP6	0.22047	6.66E-05	0.002837
ENSG00000084234	APLP2	0.21457	3.93E-06	0.00022173
ENSG00000071054	MAP4K4	0.21149	8.52E-05	0.0035044
ENSG00000198938	MT-CO3	0.15391	0.00056793	0.018727
ENSG00000167680	SEMA6B	0.13389	4.10E-05	0.0018246
ENSG00000184009	ACTG1	0.10681	0.00084956	0.026206

Table S2. Downregulated genes in YAC-treated iEPCs

Gene_id	hgnc_symbol	log2.Fold_change	*p* value	q value
ENSG00000166743	ACSM1	-6.2954	5.68E-05	0.0024483
ENSG00000005187	ACSM3	-4.6727	1.18E-27	3.82E-25
ENSG00000150471	ADGRL3	-4.4106	0.00029303	0.010531
ENSG00000078401	EDN1	-4.0839	3.51E-09	3.04E-07
ENSG00000130294	KIF1A	-3.4095	0.00074421	0.02345
ENSG00000166033	HTRA1	-3.2916	4.89E-06	0.0002684
ENSG00000118523	CTGF	-3.0745	3.11E-36	1.59E-33
ENSG00000128645	HOXD1	-3.0708	4.89E-10	4.84E-08
ENSG00000164283	ESM1	-3.0093	2.27E-115	3.85E-112
ENSG00000105825	TFPI2	-3.0026	1.01E-35	4.95E-33
ENSG00000138623	SEMA7A	-2.9759	4.82E-14	7.46E-12
ENSG00000100311	PDGFB	-2.9547	1.40E-30	5.60E-28
ENSG00000144476	ACKR3	-2.92	2.33E-75	2.56E-72
ENSG00000142871	CYR61	-2.9119	2.25E-31	9.88E-29
ENSG00000131477	RAMP2	-2.8801	4.13E-05	0.0018321
ENSG00000076716	GPC4	-2.8164	6.91E-07	4.43E-05
ENSG00000198053	SIRPA	-2.8141	2.61E-07	1.79E-05
ENSG00000152092	ASTN1	-2.7434	2.88E-06	0.0001662
ENSG00000113083	LOX	-2.7208	8.21E-09	6.81E-07
ENSG00000174059	CD34	-2.6711	7.32E-37	3.83E-34
ENSG00000162654	GBP4	-2.6609	4.57E-08	3.43E-06
ENSG00000172201	ID4	-2.6601	1.37E-05	0.00068033
ENSG00000166257	SCN3B	-2.628	2.53E-17	4.76E-15
ENSG00000165507	DEPP1	-2.6092	2.72E-30	1.05E-27
ENSG00000102924	CBLN1	-2.5643	0.0002039	0.0076073
ENSG00000148677	ANKRD1	-2.5076	3.48E-11	3.88E-09
ENSG00000143869	GDF7	-2.4572	3.06E-11	3.45E-09
ENSG00000160111	CPAMD8	-2.4204	0.00025658	0.009343
ENSG00000117152	RGS4	-2.411	0.00024845	0.0090771
ENSG00000136383	ALPK3	-2.4108	3.87E-36	1.93E-33
ENSG00000127533	F2RL3	-2.3908	2.98E-12	3.83E-10
ENSG00000205978	NYNRIN	-2.3393	3.99E-07	2.67E-05
ENSG00000105290	APLP1	-2.326	3.77E-05	0.0017191
ENSG00000162591	MEGF6	-2.2777	1.46E-41	8.22E-39
ENSG00000141448	GATA6	-2.2744	2.72E-06	0.00015765

ENSG00000065717	TLE2	-2.2565	8.39E-25	2.31E-22
ENSG00000124225	PMEPA1	-2.1214	2.84E-13	4.14E-11
ENSG00000157404	KIT	-2.0778	2.04E-06	0.0001206
ENSG00000079308	TNS1	-2.054	0.00088036	0.027005
ENSG00000122863	CHST3	-2.0536	7.99E-05	0.0033138
ENSG00000119630	PGF	-2.0382	1.33E-75	1.54E-72
ENSG00000149591	TAGLN	-2.0219	8.86E-05	0.0036282
ENSG00000121904	CSMD2	-2.0171	0.0011317	0.033727
ENSG00000225968	ELFN1	-1.9802	0.00011776	0.0046922
ENSG00000101335	MYL9	-1.9768	1.27E-05	0.00063056
ENSG00000198535	C2CD4A	-1.9764	3.58E-05	0.0016451
ENSG00000171864	PRND	-1.9466	5.56E-62	5.32E-59
ENSG00000147852	VLDLR	-1.9354	1.01E-13	1.52E-11
ENSG00000140479	PCSK6	-1.9211	4.25E-07	2.82E-05
ENSG00000026508	CD44	-1.8962	5.05E-10	4.98E-08
ENSG00000023171	GRAMD1B	-1.8721	4.63E-05	0.0020382
ENSG00000180921	FAM83H	-1.8694	3.02E-05	0.0014042
ENSG00000011028	MRC2	-1.8247	7.51E-13	1.05E-10
ENSG00000143878	RHOB	-1.8181	3.81E-109	5.59E-106
ENSG00000128594	LRRC4	-1.8087	6.62E-11	7.03E-09
ENSG00000143375	CGN	-1.7908	1.01E-06	6.20E-05
ENSG00000145147	SLIT2	-1.7856	2.29E-28	7.50E-26
ENSG00000169604	ANTXR1	-1.7849	7.48E-05	0.0031169
ENSG00000146592	CREB5	-1.7802	9.37E-05	0.0038018
ENSG00000100368	CSF2RB	-1.7775	2.32E-05	0.0011017
ENSG00000224189	HAGLR	-1.7458	0.00025965	0.0094392
ENSG00000183098	GPC6	-1.7389	0.00051671	0.017297
ENSG00000087245	MMP2	-1.6767	2.25E-46	1.65E-43
ENSG00000106366	SERPINE1	-1.6602	4.62E-45	3.18E-42
ENSG00000183087	GAS6	-1.6325	0.00098432	0.029861
ENSG00000166016	ABTB2	-1.6254	2.29E-12	2.98E-10
ENSG00000114019	AMOTL2	-1.622	4.44E-13	6.30E-11
ENSG00000162512	SDC3	-1.6215	1.45E-13	2.15E-11
ENSG00000160145	KALRN	-1.6099	3.04E-06	0.00017345
ENSG00000182752	PAPPA	-1.609	6.92E-05	0.0029221
ENSG00000106484	MEST	-1.5955	1.40E-05	0.00069004
ENSG00000171388	APLN	-1.5867	6.12E-48	4.64E-45

ENSG00000164327	RICTOR	-1.5801	1.57E-13	2.32E-11
ENSG00000172183	ISG20	-1.5764	0.00041888	0.014508
ENSG00000136603	SKIL	-1.5707	0.0012403	0.036765
ENSG00000172985	SH3RF3	-1.5579	0.00088676	0.027051
ENSG00000125872	LRRN4	-1.5475	2.15E-06	0.00012644
ENSG00000124920	MYRF	-1.5443	5.44E-06	0.00029566
ENSG00000129116	PALLD	-1.5382	1.01E-10	1.05E-08
ENSG00000130300	PLVAP	-1.538	9.00E-246	6.60E-242
ENSG00000080573	COL5A3	-1.5196	4.65E-31	1.97E-28
ENSG00000129038	LOXL1	-1.5136	2.06E-05	0.00099158
ENSG00000174938	SEZ6L2	-1.4798	2.45E-06	0.00014233
ENSG00000159388	BTG2	-1.452	2.19E-07	1.52E-05
ENSG00000092096	SLC22A17	-1.4503	9.89E-06	0.00050099
ENSG00000138411	HECW2	-1.4429	7.39E-05	0.0030871
ENSG00000153443	UBALD1	-1.4313	6.83E-07	4.41E-05
ENSG00000146094	DOK3	-1.4242	1.46E-06	8.73E-05
ENSG00000138650	PCDH10	-1.4205	1.27E-27	4.05E-25
ENSG00000197256	KANK2	-1.4026	6.40E-18	1.26E-15
ENSG00000127415	IDUA	-1.3866	5.16E-05	0.0022379
ENSG00000144749	LRIG1	-1.3865	0.00021737	0.0080485
ENSG00000083857	FAT1	-1.3678	2.04E-11	2.40E-09
ENSG00000196739	COL27A1	-1.3607	9.89E-11	1.04E-08
ENSG00000166963	MAP1A	-1.3499	0.0001519	0.0058205
ENSG00000101670	LIPG	-1.3407	6.10E-14	9.32E-12
ENSG00000101160	CTSZ	-1.3264	3.34E-11	3.75E-09
ENSG00000113657	DPYSL3	-1.3166	0.00030667	0.010932
ENSG00000019144	PHLDB1	-1.2938	6.34E-18	1.26E-15
ENSG00000170421	KRT8	-1.289	2.00E-27	6.03E-25
ENSG00000089820	ARHGAP4	-1.2863	1.23E-12	1.65E-10
ENSG00000010319	SEMA3G	-1.2828	1.39E-27	4.36E-25
ENSG00000178860	MSC	-1.2739	2.21E-05	0.0010522
ENSG00000150938	CRIM1	-1.2646	1.35E-98	1.75E-95
ENSG00000110844	PRPF40B	-1.2587	0.0011924	0.035439
ENSG00000124006	OBSL1	-1.2453	6.14E-05	0.0026271
ENSG00000128591	FLNC	-1.2435	3.62E-11	4.03E-09
ENSG00000151967	SCHIP1	-1.2302	0.00073359	0.023182
ENSG00000132718	SYT11	-1.2279	1.73E-05	0.000842

ENSG00000134243	SORT1	-1.2113	1.63E-10	1.66E-08
ENSG00000175155	YPEL2	-1.2075	0.00056375	0.018617
ENSG00000138448	ITGAV	-1.2065	1.89E-15	3.27E-13
ENSG00000152137	HSPB8	-1.1922	0.00098669	0.029891
ENSG00000076356	PLXNA2	-1.1795	3.71E-19	8.16E-17
ENSG00000123094	RASSF8	-1.1706	0.00037418	0.013105
ENSG00000139263	LRIG3	-1.1671	1.16E-08	9.41E-07
ENSG00000105426	PTPRS	-1.1658	9.32E-05	0.0037894
ENSG00000130635	COL5A1	-1.1599	3.10E-46	2.20E-43
ENSG00000100234	TIMP3	-1.1471	6.08E-24	1.63E-21
ENSG00000196628	TCF4	-1.1467	3.93E-13	5.65E-11
ENSG00000079337	RAPGEF3	-1.1381	1.35E-06	8.17E-05
ENSG00000140545	MFGE8	-1.1355	1.96E-09	1.74E-07
ENSG00000163739	CXCL1	-1.1344	0.00069432	0.022132
ENSG00000166147	FBN1	-1.126	1.19E-09	1.10E-07
ENSG00000143322	ABL2	-1.1139	3.26E-08	2.50E-06
ENSG00000005469	CROT	-1.1098	4.80E-06	0.00026482
ENSG00000103257	SLC7A5	-1.1062	0.0014081	0.041075
ENSG00000182492	BGN	-1.1043	3.18E-25	8.85E-23
ENSG00000113555	PCDH12	-1.1033	9.76E-19	2.10E-16
ENSG00000159176	CSRP1	-1.0922	3.06E-18	6.11E-16
ENSG00000153162	BMP6	-1.0908	1.40E-12	1.84E-10
ENSG00000034677	RNF19A	-1.0798	6.11E-07	3.98E-05
ENSG00000129422	MTUS1	-1.0782	1.36E-09	1.24E-07
ENSG00000128567	PODXL	-1.0707	2.72E-11	3.12E-09
ENSG00000159658	EFCAB14	-1.0631	1.04E-32	4.66E-30
ENSG00000123989	CHPF	-1.0618	0.0001002	0.0040509
ENSG00000166508	MCM7	-1.058	0.00014341	0.0055144
ENSG00000187498	COL4A1	-1.0569	2.91E-221	1.60E-217
ENSG00000110811	P3H3	-1.0566	3.39E-06	0.00019244
ENSG00000145681	HAPLN1	-1.056	1.91E-25	5.38E-23
ENSG00000137831	UACA	-1.0541	6.56E-09	5.51E-07
ENSG00000150995	ITPR1	-1.0401	0.00016986	0.0064191
ENSG00000132688	NES	-1.0323	5.14E-42	2.97E-39
ENSG00000075420	FNDC3B	-1.0297	3.94E-15	6.66E-13
ENSG00000175264	CHST1	-1.0291	3.37E-43	2.12E-40
ENSG00000143641	GALNT2	-1.0287	1.39E-11	1.66E-09

ENSG00000173530	TNFRSF10D	-1.0233	1.51E-22	3.82E-20
ENSG00000183496	MEX3B	-1.0222	0.00011292	0.0045157
ENSG00000163297	ANTXR2	-1.0082	4.40E-07	2.91E-05
ENSG00000169105	CHST14	-1.0072	0.0010736	0.03217
ENSG00000158859	ADAMTS4	-1.0059	0.00041358	0.01437
ENSG00000107731	UNC5B	-0.9979	3.55E-50	3.01E-47
ENSG00000006451	RALA	-0.9969	7.73E-13	1.08E-10
ENSG00000168487	BMP1	-0.99628	9.97E-07	6.17E-05
ENSG00000134871	COL4A2	-0.99566	1.40E-182	5.15E-179
ENSG00000108821	COL1A1	-0.98891	1.65E-14	2.69E-12
ENSG00000179776	CDH5	-0.98085	3.50E-123	7.08E-120
ENSG00000140416	TPM1	-0.97968	2.12E-09	1.87E-07
ENSG00000122786	CALD1	-0.9778	8.53E-13	1.16E-10
ENSG00000161791	FMNL3	-0.97256	1.69E-16	3.02E-14
ENSG00000163346	PBXIP1	-0.96609	1.34E-08	1.08E-06
ENSG00000149428	HYOU1	-0.96607	1.72E-17	3.28E-15
ENSG00000112290	WASF1	-0.95007	0.00024519	0.008988
ENSG00000076706	MCAM	-0.94897	2.93E-122	5.37E-119
ENSG00000142961	MOB3C	-0.94853	5.12E-05	0.0022239
ENSG00000133466	C1QTNF6	-0.94399	4.30E-06	0.00023811
ENSG00000154277	UCHL1	-0.93786	2.09E-19	4.68E-17
ENSG00000130309	COLGALT1	-0.92572	1.67E-27	5.10E-25
ENSG00000115414	FN1	-0.92137	8.69E-164	2.73E-160
ENSG00000184557	SOCS3	-0.92096	8.82E-05	0.0036175
ENSG00000139112	GABARAPL1	-0.91924	0.00031183	0.011098
ENSG00000220785	MTMR9LP	-0.91619	0.0010335	0.031138
ENSG00000160058	BSDC1	-0.91348	0.00028256	0.010171
ENSG00000154096	THY1	-0.90837	2.35E-05	0.0011126
ENSG00000099814	CEP170B	-0.9083	3.35E-05	0.0015446
ENSG00000107438	PDLIM1	-0.90497	0.0013814	0.040352
ENSG00000123384	LRP1	-0.89839	4.17E-06	0.00023203
ENSG00000171992	SYNPO	-0.89516	8.31E-08	6.09E-06
ENSG00000115380	EFEMP1	-0.89275	3.22E-08	2.48E-06
ENSG00000221968	FADS3	-0.89094	6.37E-06	0.00033996
ENSG00000007384	RHBDF1	-0.87863	6.65E-07	4.30E-05
ENSG00000125810	CD93	-0.86788	3.54E-123	7.08E-120
ENSG00000083444	PLOD1	-0.86596	3.47E-43	2.12E-40

ENSG00000060718	COL11A1	-0.85375	0.0007321	0.023168
ENSG00000165757	JCAD	-0.85247	1.55E-07	1.10E-05
ENSG00000099139	PCSK5	-0.84745	0.0010006	0.030271
ENSG00000164659	KIAA1324L	-0.8425	2.17E-06	0.00012713
ENSG00000173801	JUP	-0.84029	2.56E-31	1.11E-28
ENSG00000173950	XXYLT1	-0.83469	0.00018832	0.0070683
ENSG00000139514	SLC7A1	-0.82715	0.00014242	0.0055052
ENSG00000128965	CHAC1	-0.82524	0.00045585	0.015592
ENSG00000172638	EFEMP2	-0.81978	0.0012027	0.035697
ENSG00000198668	CALM1	-0.81415	7.35E-31	3.05E-28
ENSG00000142798	HSPG2	-0.81217	0	0
ENSG00000156453	PCDH1	-0.80181	3.02E-19	6.72E-17
ENSG00000196535	MYO18A	-0.80143	0.00022019	0.0081394
ENSG00000106624	AEBP1	-0.80053	0.00027757	0.010024
ENSG00000170801	HTRA3	-0.799	4.48E-10	4.46E-08
ENSG00000114554	PLXNA1	-0.79818	1.56E-07	1.10E-05
ENSG00000114541	FRMD4B	-0.78653	0.00046652	0.015883
ENSG00000158270	COLEC12	-0.78315	3.81E-08	2.89E-06
ENSG00000164683	HEY1	-0.78264	4.10E-11	4.51E-09
ENSG00000116991	SIPA1L2	-0.78226	1.33E-07	9.56E-06
ENSG00000115457	IGFBP2	-0.78139	8.29E-56	7.60E-53
ENSG00000168036	CTNNB1	-0.77595	5.21E-13	7.35E-11
ENSG00000184588	PDE4B	-0.77406	0.0017382	0.049076
ENSG00000136560	TANK	-0.77313	0.00036846	0.012927
ENSG00000106070	GRB10	-0.77232	9.27E-10	8.68E-08
ENSG00000185215	TNFAIP2	-0.76243	0.00029769	0.010663
ENSG00000136156	ITM2B	-0.76232	3.25E-16	5.76E-14
ENSG00000150907	FOXO1	-0.76214	0.00010094	0.0040737
ENSG00000173598	NUDT4	-0.75926	1.32E-28	4.62E-26
ENSG00000111057	KRT18	-0.75807	0.0010888	0.032581
ENSG00000111897	SERINC1	-0.74881	5.30E-05	0.0022949
ENSG00000071246	VASH1	-0.74072	2.94E-23	7.80E-21
ENSG00000142949	PTPRF	-0.7406	2.17E-08	1.71E-06
ENSG00000088387	DOCK9	-0.73736	5.91E-10	5.70E-08
ENSG00000130508	PXDN	-0.73693	5.21E-77	6.37E-74
ENSG00000151233	GXYLT1	-0.73455	0.00057195	0.018826
ENSG00000197077	KIAA1671	-0.73215	0.00011242	0.0045036

ENSG00000154133	ROBO4	-0.72767	9.56E-08	6.97E-06
ENSG00000141696	P3H4	-0.72734	8.30E-06	0.00043067
ENSG00000118946	PCDH17	-0.72224	0.00042941	0.014827
ENSG00000112769	LAMA4	-0.72007	1.57E-05	0.00077109
ENSG00000158352	SHROOM4	-0.70798	0.0010094	0.030494
ENSG00000166086	JAM3	-0.70641	2.16E-11	2.52E-09
ENSG00000145050	MANF	-0.70563	0.00058916	0.019283
ENSG00000058668	ATP2B4	-0.70458	1.14E-08	9.32E-07
ENSG00000103855	CD276	-0.69081	1.90E-11	2.25E-09
ENSG00000133872	SARAF	-0.68784	1.65E-05	0.00080313
ENSG00000011422	PLAUR	-0.68533	0.00034977	0.012348
ENSG00000187513	GJA4	-0.68038	1.47E-28	5.05E-26
ENSG00000114251	WNT5A	-0.67833	0.00025633	0.009343
ENSG00000136026	CKAP4	-0.66961	2.24E-08	1.75E-06
ENSG00000136717	BIN1	-0.66711	1.88E-05	0.00090711
ENSG00000109501	WFS1	-0.66407	4.50E-05	0.0019855
ENSG00000120278	PLEKHG1	-0.66062	1.41E-07	1.01E-05
ENSG00000129128	SPCS3	-0.646	0.00077358	0.024237
ENSG00000167566	NCKAP5L	-0.64162	3.93E-05	0.0017829
ENSG00000161638	ITGA5	-0.63762	1.53E-41	8.41E-39
ENSG00000173535	TNFRSF10C	-0.63117	1.51E-05	0.00074094
ENSG00000198467	TPM2	-0.62297	0.00011801	0.0046933
ENSG00000145911	N4BP3	-0.62121	6.82E-12	8.43E-10
ENSG00000137962	ARHGAP29	-0.61891	7.15E-07	4.57E-05
ENSG00000082781	ITGB5	-0.61836	2.84E-05	0.0013289
ENSG00000008517	IL32	-0.61175	0.00012026	0.0047744
ENSG00000144668	ITGA9	-0.60677	4.03E-06	0.00022534
ENSG00000143772	ITPKB	-0.60109	6.12E-05	0.002623
ENSG00000186111	PIP5K1C	-0.60094	4.83E-05	0.0021215
ENSG00000104331	IMPAD1	-0.58806	0.0016642	0.047411
ENSG00000171680	PLEKHG5	-0.5874	0.00011421	0.004559
ENSG00000068001	HYAL2	-0.58564	1.58E-08	1.26E-06
ENSG00000112655	PTK7	-0.58243	0.00018582	0.0069861
ENSG00000175274	TP53I11	-0.58062	2.49E-11	2.87E-09
ENSG00000169756	LIMS1	-0.57638	3.28E-05	0.0015143
ENSG00000140943	MBTPS1	-0.57445	0.00065466	0.021081
ENSG00000161671	EMC10	-0.57317	6.49E-06	0.00034566

ENSG00000158710	TAGLN2	-0.57188	2.12E-07	1.48E-05
ENSG00000118495	PLAGL1	-0.5695	1.88E-05	0.00090711
ENSG00000164877	MICALL2	-0.56777	0.0016976	0.048143
ENSG00000074181	NOTCH3	-0.56612	2.83E-05	0.0013257
ENSG00000162458	FBLIM1	-0.56378	1.98E-10	2.01E-08
ENSG00000121966	CXCR4	-0.55882	5.03E-11	5.45E-09
ENSG00000196923	PDLIM7	-0.55708	5.19E-07	3.42E-05
ENSG00000171316	CHD7	-0.55501	0.00082878	0.025637
ENSG00000205542	TMSB4X	-0.55253	1.62E-25	4.62E-23
ENSG00000135723	FHOD1	-0.54209	0.0011174	0.033347
ENSG00000117298	ECE1	-0.542	1.29E-25	3.73E-23
ENSG00000141429	GALNT1	-0.5396	6.21E-05	0.0026517
ENSG00000149257	SERPINH1	-0.53834	5.81E-14	8.94E-12
ENSG00000125844	RRBP1	-0.53752	7.35E-06	0.00038785
ENSG00000099250	NRP1	-0.53422	7.78E-12	9.56E-10
ENSG00000011105	TSPAN9	-0.53374	8.43E-05	0.0034735
ENSG00000159363	ATP13A2	-0.52784	3.98E-06	0.00022317
ENSG00000149564	ESAM	-0.52708	7.18E-17	1.32E-14
ENSG00000136490	LIMD2	-0.5244	4.03E-05	0.0018086
ENSG00000104341	LAPTM4B	-0.52287	2.79E-07	1.91E-05
ENSG00000064042	LIMCH1	-0.52073	0.00016131	0.0061169
ENSG00000146083	RNF44	-0.51957	0.0017595	0.049486
ENSG00000120318	ARAP3	-0.51158	7.34E-06	0.00038785
ENSG00000185361	TNFAIP8L1	-0.51015	8.05E-06	0.00041949
ENSG00000077782	FGFR1	-0.5096	3.44E-08	2.63E-06
ENSG00000120889	TNFRSF10B	-0.50328	1.71E-06	0.00010202
ENSG00000164736	SOX17	-0.50011	7.90E-07	4.99E-05
ENSG00000106351	AGFG2	-0.49825	0.0017255	0.048781
ENSG00000112697	TMEM30A	-0.49634	8.36E-06	0.00043268
ENSG00000179044	EXOC3L1	-0.49272	0.00018127	0.0068266
ENSG00000143384	MCL1	-0.49248	0.00045309	0.015522
ENSG00000092841	MYL6	-0.49018	9.80E-07	6.09E-05
ENSG00000145632	PLK2	-0.48493	1.09E-05	0.00055036
ENSG00000148700	ADD3	-0.4802	0.00023255	0.0085388
ENSG00000128512	DOCK4	-0.47676	0.00064827	0.020937
ENSG00000198844	ARHGEF15	-0.47632	0.0014496	0.042061
ENSG00000168758	SEMA4C	-0.47602	2.24E-06	0.00013074

ENSG00000069020	MAST4	-0.47268	0.00013884	0.0053763
ENSG00000173482	PTPRM	-0.4663	7.77E-05	0.0032315
ENSG00000118680	MYL12B	-0.4641	0.00036798	0.012927
ENSG00000166341	DCHS1	-0.46364	1.16E-08	9.44E-07
ENSG00000167552	TUBA1A	-0.46339	3.27E-08	2.50E-06
ENSG00000135916	ITM2C	-0.46299	1.59E-18	3.29E-16
ENSG00000168268	NT5DC2	-0.45864	0.00060489	0.01971
ENSG00000084207	GSTP1	-0.45585	5.99E-05	0.0025751
ENSG00000133026	MYH10	-0.45033	0.00040687	0.014182
ENSG00000138829	FBN2	-0.44863	0.00067302	0.021515
ENSG00000003436	TFPI	-0.4455	4.87E-05	0.0021301
ENSG00000091136	LAMB1	-0.44501	1.04E-08	8.54E-07
ENSG00000077238	IL4R	-0.43572	0.00097779	0.029745
ENSG00000033100	CHPF2	-0.43471	0.00054022	0.017975
ENSG00000170759	KIF5B	-0.43409	2.29E-05	0.0010899
ENSG00000128917	DLL4	-0.42914	2.07E-14	3.30E-12
ENSG00000130479	MAP1S	-0.42799	0.00055448	0.018394
ENSG00000136068	FLNB	-0.42639	1.62E-08	1.29E-06
ENSG00000107957	SH3PXD2A	-0.42636	0.0001529	0.0058486
ENSG00000185112	FAM43A	-0.4232	6.76E-05	0.0028698
ENSG00000070610	GBA2	-0.41868	0.00063547	0.020614
ENSG00000147065	MSN	-0.41845	9.46E-11	9.96E-09
ENSG00000167470	MIDN	-0.41591	3.78E-07	2.54E-05
ENSG00000128849	CGNL1	-0.41055	0.00052421	0.017522
ENSG00000127920	GNG11	-0.40973	0.00047177	0.016013
ENSG00000018408	WWTR1	-0.40614	0.0015161	0.04376
ENSG00000130787	HIP1R	-0.4014	0.00088611	0.027051
ENSG00000152291	TGOLN2	-0.40117	0.001266	0.037425
ENSG00000134531	EMP1	-0.39841	2.18E-05	0.0010418
ENSG00000110880	CORO1C	-0.39067	1.79E-05	0.00086883
ENSG00000135074	ADAM19	-0.38561	5.42E-07	3.56E-05
ENSG00000005884	ITGA3	-0.38441	0.00027488	0.00996
ENSG00000044574	HSPA5	-0.3842	2.90E-07	1.98E-05
ENSG00000183255	PTTG1IP	-0.3754	0.00051102	0.017159
ENSG00000111348	ARHGDIB	-0.37473	4.12E-05	0.0018315
ENSG00000177469	CAVIN1	-0.37217	1.31E-09	1.21E-07
ENSG00000115306	SPTBN1	-0.37165	1.78E-09	1.59E-07

ENSG00000157570	TSPAN18	-0.36865	9.49E-07	5.91E-05
ENSG00000020181	ADGRA2	-0.36628	6.23E-08	4.63E-06
ENSG00000130402	ACTN4	-0.36505	2.14E-07	1.48E-05
ENSG00000136378	ADAMTS7	-0.36404	1.04E-06	6.36E-05
ENSG00000167004	PDIA3	-0.36118	0.00010812	0.0043552
ENSG00000127511	SIN3B	-0.35624	0.00097986	0.029767
ENSG00000139644	TMBIM6	-0.3556	2.43E-05	0.0011452
ENSG00000155660	PDIA4	-0.35027	0.00032825	0.011626
ENSG00000125089	SH3TC1	-0.33289	0.00060143	0.019655
ENSG00000128052	KDR	-0.33009	4.11E-15	6.91E-13
ENSG00000143870	PDIA6	-0.32008	0.0012964	0.038274
ENSG00000127603	MACF1	-0.31512	5.71E-05	0.0024596
ENSG00000142173	COL6A2	-0.30725	0.0001207	0.0047833
ENSG00000160796	NBEAL2	-0.30586	0.00012145	0.0048043
ENSG00000006118	TMEM132A	-0.30222	9.38E-06	0.00047959
ENSG00000139567	ACVRL1	-0.28846	0.00011066	0.0044415
ENSG00000124126	PREX1	-0.27814	0.0010214	0.030817
ENSG00000179218	CALR	-0.27151	1.39E-08	1.12E-06
ENSG00000150687	PRSS23	-0.26085	3.13E-05	0.0014515
ENSG00000113140	SPARC	-0.24393	4.11E-12	5.22E-10
ENSG00000105329	TGFB1	-0.24199	1.98E-06	0.00011765
ENSG00000204301	NOTCH4	-0.23164	2.23E-06	0.0001304
ENSG00000124942	AHNAK	-0.22412	3.46E-07	2.34E-05
ENSG00000150093	ITGB1	-0.19425	8.23E-05	0.0034078
ENSG00000114353	GNAI2	-0.17623	0.00041652	0.01445
ENSG00000197746	PSAP	-0.16659	0.00086497	0.026607
ENSG00000148400	NOTCH1	-0.15439	0.00048529	0.016396
